# m^6^A RNA methylation impairs gene expression variability and reproductive thermotolerance in *Arabidopsis*

**DOI:** 10.1186/s13059-022-02814-8

**Published:** 2022-11-23

**Authors:** Ling Wang, Haiyan Zhuang, Wenwen Fan, Xia Zhang, Haihong Dong, Hongxing Yang, Jungnam Cho

**Affiliations:** 1 Shanghai Key Laboratory of Plant Functional Genomics and Resources, Shanghai Chenshan Botanical Garden, Shanghai, 201602 China; 2 National Key Laboratory of Plant Molecular Genetics, CAS Center for Excellence in Molecular Plant Sciences, Shanghai Institute of Plant Physiology and Ecology, Chinese Academy of Sciences, Shanghai, 200032 China; 3 University of Chinese Academy of Science, Beijing, 100049 China; 4 College of Life Sciences, Shanghai Normal University, Shanghai, 200234 China; 5 CAS-JIC Centre of Excellence for Plant and Microbial Science, Shanghai, 200032 China

**Keywords:** m^6^A RNA methylation, Gene expression variability, Heat tolerance, *AtALKBH10B*, *Arabidopsis thaliana*

## Abstract

Heat-imposed crop failure is often attributed to reduced thermotolerance of floral tissues; however, the underlying mechanism remains unknown. Here, we demonstrate that m^6^A RNA methylation increases in *Arabidopsis* flowers and negatively regulates gene expression variability. Stochastic gene expression provides flexibility to cope with environmental stresses. We find that reduced transcriptional fluctuation is associated with compromised activation of heat-responsive genes. Moreover, disruption of an RNA demethylase AtALKBH10B leads to lower gene expression variability, suppression of heat-activated genes, and strong reduction of plant fertility. Our work proposes a novel role for RNA methylation in the bet-hedging strategy of heat stress response.

**Supplementary Information:**

The online version contains supplementary material available at 10.1186/s13059-022-02814-8.

## Background

Reproductive tissues of plants are more susceptible to heat stress than in other developmental stages [1, 2]. Previous studies suggested that unfolded protein response factors play an important role in the reproductive thermotolerance [3–6]. For example, basic leucine zipper transcription factors, bZIP17, bZIP28, and bZIP60, are essential for maintaining fertility under heat stress [3–5]. In addition, SQUAMOSA promoter-binding protein-like (SPL) transcription factors, SPL1 and SPL12, are required for thermotolerance at the reproductive stage of *Arabidopsis* [7]. Moreover, various small RNAs were identified in the heat-stressed flowers of flax, soybean, and maize [8–10]; however, their mode of action is still elusive.

Sporadic gene expression is an important cellular feature that increases the adaptability of an organism to changing environments [11–13]. In unicellular organisms, noisy transcription enhances fitness in adverse growth conditions [11, 14–16]. Stochastic gene expression is also observed in multicellular organisms; for instance, clonal heterogeneity of gene expression is important for lineage choice of mouse hematopoietic progenitor cells [17], the intestinal differentiation of nematode is also influenced by gene expression variability [18], and a robust transcription of NF-κB is attributed to both intrinsic and extrinsic variability [19]. In *Arabidopsis*, it was previously suggested that genes with variable expression were enriched with factors involved in stress responses [20–23]. Therefore, noisy gene expression is an evolutionary conserved and biologically relevant mechanism that enhances flexibility of cellular responses.

In this study, we show that N6-methyladenosine (m^6^A), a widespread RNA modification in mRNAs [24–26], is strongly increased in *Arabidopsis* flowers and inversely correlated with gene expression variability, which then compromises reproductive thermotolerance. Our work suggests a novel role for RNA methylation and gene expression variability in heat tolerance, which can help mitigate the heat-imposed reproductive failure of crop plants.

## Results and discussion

Plants’ resilience to environmental challenges varies during development and floral tissues are particularly more sensitive to heat stress [1, 2]. Such heat susceptibility during the reproductive development can cause the reduction of yield and quality of fruit and cereal crops. To understand the molecular mechanisms underlying the heat-imposed reproductive failure in *Arabidopsis*, we carried out transcriptomic analyses of the heat-stressed flower and leaf samples and found that flowers exhibited a distinct pattern of gene expression under heat stress (Additional file 1: Fig. S1). Genes in clusters 1 and 10, for instance, were strongly activated upon heat treatment in leaves, while in flowers, their expression levels were not altered dramatically (Additional file 1: Fig. S1). Unfortunately, the fundamental mechanisms driving such divergence of heat responsiveness in different tissues are not well understood.

**Fig. 1:**
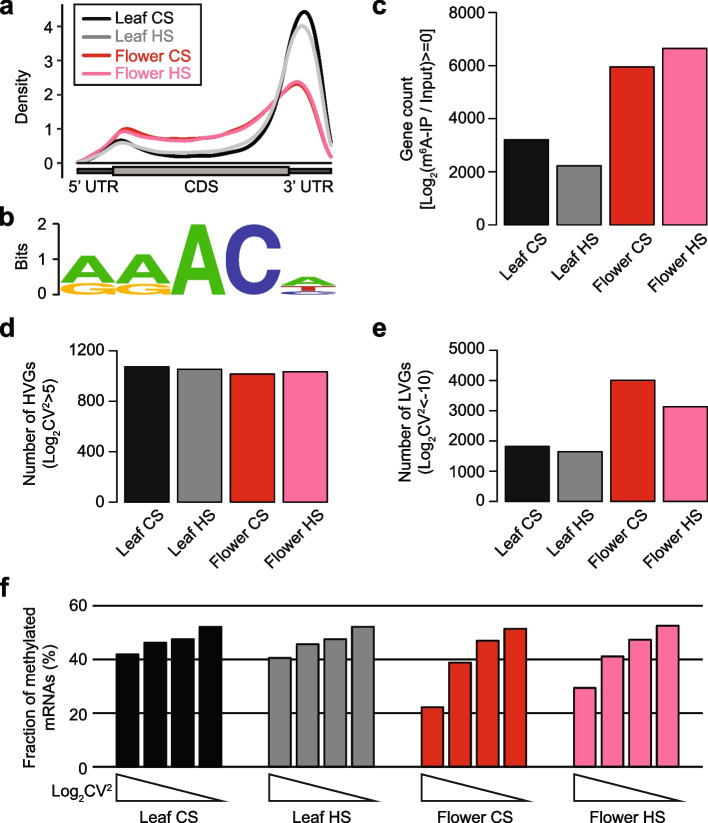
RNA methylation and expression variability is inversely correlated. **a** Density distribution of m^6^A in the control and heat-stressed leaves and flowers of *Arabidopsis*. UTR and CDS are marked as dark and light grey boxes, respectively. CS, control sample; HS, heat-stressed sample. **b** Consensus sequence motif of m^6^A-modified sites in the non-stressed leaf sample. The top 1000 most m^6^A-enriched regions were used for the analysis. **c** Number of genes with m^6^A enrichment score greater than 0. Enrichment of m^6^A was determined by log2-converted fold change of m^6^A-immunoprecipitated to input RNA levels. **d**, **e** Number of HVGs (**d**) and LVGs (**e**) in leaves and flowers subjected to the control and heat stress treatments. HVGs and LVGs are genes with log_2_CV^2^ greater than 5 and lower than − 10, respectively. CV^2^ refers to coefficient of variation of a gene which was determined by FPKMs of RNA-seq performed for three biological replications. **f** Fraction of m^6^A-modified transcripts in quartiles of gene expression variability. Methylated transcript was as defined in **c**

Previous studies suggested that RNA modifications (often referred to as epitranscriptomic marks) are implicated in cellular response to environmental challenges [27–29]; however, the functional roles of RNA modification in the heat stress response of *Arabidopsis* remain largely unknown. In order to test if the developmentally divergent heat response can be attributed to m^6^A, the most widespread RNA modification type in mRNA, we carried out m^6^A-RNA immunoprecipitation (RIP)-seq experiments using the same samples tested in Fig. S1 (Fig. 1a and b; Additional file 1: Fig. S2 and S3). Unlike the vast changes of gene expression levels observed in Fig. S1, the m^6^A levels did not show any dramatic difference in the heat stress condition and between leaves and flowers (Additional file 1: Fig. S4). We then measured the m^6^A enrichment (determined by fold change of m^6^A-immunoprecipitated to total RNA) and found that the gene expression levels were poorly correlated with the m^6^A enrichment (Additional file 1: Fig. S5). In addition, Pearson correlation coefficient analysis revealed that heat stress-induced gene expression and m^6^A enrichment changes are only marginally correlated (Additional file 1: Fig. S6). This is in agreement with previous studies that suggested a negligible effect of RNA methylation in transcriptome-wide mRNA steady-state levels in *Arabidopsis* [30]. Taken together, heat stress causes distinct transcriptomic changes in the *Arabidopsis* leaves and flowers and triggers little changes of m^6^A levels.

Our m^6^A RNA methylation data revealed that the flower samples display distinct m^6^A pattern from leaves exhibiting higher levels along the CDS and lower levels around the 3′ UTR (Fig. 1a). More importantly, a remarkably stronger enrichment of m^6^A was detected in flowers (Fig. 1c; Additional file 1: Fig. S7), which is consistent with previous studies [31, 32]. We therefore hypothesized that strong m^6^A enrichment in flowers might play a role in the reproductive thermosensitivity by modulating certain RNA processes. Intriguingly, we found a strong negative correlation between m^6^A enrichment and variability of gene expression (Fig. 1d–f). Gene expression variability refers to stochastic and noisy expression of genetically identical cells and can be determined by the squared coefficient of variation (CV^2^) that is a measure of dispersion of data points. In this study, we generated RNA-seq datasets for three independent biological replicates and interestingly, genes with invariable expression in biological replications significantly overlapped with those invariable genes identified from single-cell (sc) and single-plant RNA-seq datasets (Additional file 1: Fig. S8; Additional file 2: Table S1). This suggests that biological replications can be used to infer gene expression variability.

As shown in Fig. 1d and e, while highly variable genes (HVGs) were found similar in leaves and flowers, lowly variable genes (LVGs) were in greater numbers in flowers where stronger m^6^A enrichment is observed. Figure 1f also shows that transcripts with low gene expression variability contain more methylated mRNAs. The negative correlation of gene expression variability and m^6^A levels was also observed for transcripts with higher number of m^6^A peaks which display low expression variability (Additional file 1: Fig. S9; Additional file 3: Table S2). We also examined the m^6^A levels of HVGs and LVGs determined from other datasets. In the study of Cortijo et al., HVGs and LVGs were identified from inter-individual expression variability of *Arabidopsis* [23], and LVGs contained more m^6^A-methylated transcripts (Additional file 1: Fig. S10). Similar result was also found for HVGs and LVGs identified from a scRNA-seq dataset. We analyzed a previously published scRNA-seq data generated from *Arabidopsis* root tip [33] and determined both HVGs and LVGs. Similarly, LVGs contained more m^6^A-modified transcripts than HVGs (Additional file 1: Fig. S10). It is also worth noting that the LVGs found in flowers were strongly enriched with genes involved in abiotic stress response (Additional file 1: Fig. S11), which partly indicates a functional association between expression variability and stress resistance. In conclusion, the increased RNA methylation in flowers is associated with lower gene expression variability.

**Fig. 2:**
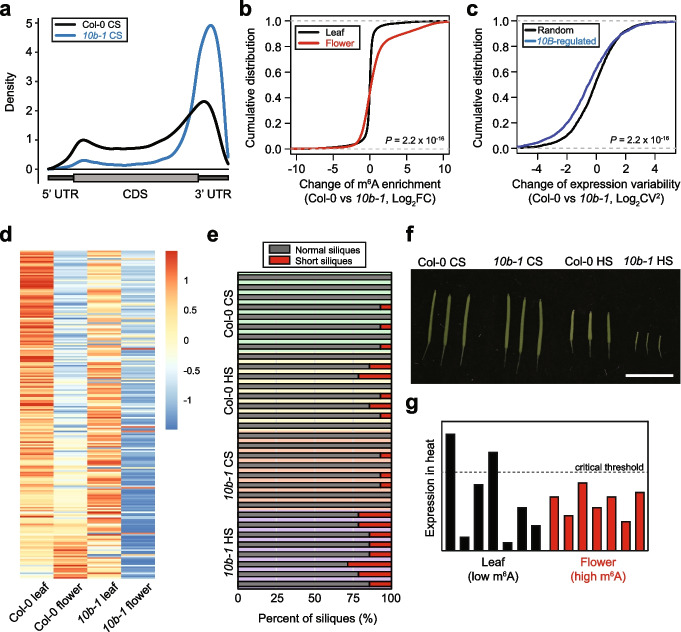
*AtALKBH10b* is required for reproductive thermotolerance. **a** Density distribution of m^6^A in the wt and *10b-1* mutant flowers. UTR and CDS are marked as dark and light grey boxes, respectively. CS, non-stressed control sample. **b** Cumulative distribution of the m^6^A enrichment fold change in *10b-1* normalized against Col-0. *P* value was obtained by the one-tailed Wilcoxon rank sum test. **c** Cumulative distribution of expression variability difference between wt and *10b-1* determined for the *10B*-regulated and randomly selected transcripts. The *10B*-regulated genes are those with at least two-fold increase of m^6^A enrichment in *10b-1* compared to wt. *P* value was obtained by the one-tailed Wilcoxon rank sum test. **d** Heatmap of FC expression upon heat stress in different samples. Genes upregulated by the heat treatment in wt leaves by at least two-fold were selected. **e** Fraction of normal and short siliques of plants subjected to the control and heat stress treatments. CS, control sample; HS, heat-stressed sample. Siliques that are longer than 10 mm were considered normal and those shorter than 10 mm were counted as short siliques. **f** A representative image for siliques of the non-stressed and heat-stressed wt and *10b-1* plants. Bar is 10 mm. **g** Schematic illustration of m^6^A-associated gene expression variability. Bars represent the level of gene expression of individual samples in heat. Floral tissues exhibit stronger m^6^A RNA methylation and lower expression variability. Dashed line indicates a critical threshold required for heat tolerance. Reduced expression variability in flowers diminishes the gene activation to a level below the critical point

In search of possible regulators of m^6^A in *Arabidopsis* flower, we focused on *AtALKBH10B* (hereinafter *10B*) because its expression level is the highest in the floral tissue among the five RNA demethylases encoded in the *Arabidopsis* genome (Additional file 1: Fig. S12). To test if *10B* plays a role in the m^6^A-associated gene expression variability, we generated the m^6^A-RIP-seq and RNA-seq data using the *10b-1* mutant (Additional file 1: Fig. S13). In the m^6^A enrichment analyses, we found only limited number of transcripts with altered m^6^A levels in the leaf sample of *10b-1* (Additional file 4: Table S3) and were unable to observe any significant differences in m^6^A distribution between the wild type (wt) and *10b-1* leaves (Additional file 1: Fig. S14). However, the flower sample of *10b-1* exhibited distinct m^6^A distribution as compared with wt, showing stronger peak around the stop codon and weaker enrichment along the CDS (Fig. 2a). Most importantly, the m^6^A enrichment level was greatly increased in the flowers of *10b-1*, while in leaves the difference of RNA methylation was only marginal (Fig. 2b; Additional file 4: Table S3), which collectively suggests that 10B mediates RNA demethylation mostly in flowers of *Arabidopsis*. We thus assessed the gene expression variability of the *10B*-regulated genes, which were defined as those with increased RNA methylation in the *10b-1* mutant. Interestingly, we found that the *10B*-regulated transcripts exhibit reduced level of gene expression variability as compared with randomly selected mRNAs (Fig. 2c; Additional file 1: Fig. S15). These data further support the notion that increased m^6^A RNA methylation leads to reduction in gene expression variability.

Previously, several studies proposed that stochastic gene transcription can be dictated by promoter structure, DNA methylation, and chromatin status [16, 23, 34]. Further to the control at DNA or chromatin levels, we showed that an epitranscriptomic mark adds to the complex regulation of gene expression variability. Given that m^6^A-methylated mRNAs are preferably located to cytoplasmic stress granules (SGs) [27, 35–37], we speculated that the physical confinement of m^6^A-modified transcripts to SGs might account for the invariability of gene expression. To test this possibility, we compared LVGs and random genes for their SG enrichment score determined in our previous study [38], and LVGs indeed showed stronger SG enrichment (Additional file 1: Fig. S16). However, the precise mechanisms as to how low gene expression variability is attributed to RNA sequestration to SGs require further investigation.

Lastly, in order to understand the functional relevance of gene expression variability in the heat stress response of *Arabidopsis*, we examined the heat responsiveness of genes in leaves and flowers of wt and *10b-1*. For this, the heat-activated genes in the wt leaves were selected and their fold change expression in heat was compared. Noticeably, the fold change of heat-activated genes was drastically compromised in the wt flowers, and it was further reduced in the *10b-1* flowers (Fig. 2d). Importantly, the *10b-1* mutant plants exhibited more severe fertility defects in the heat stress condition (Fig. 2e and f), indicating that *10B* is required for heat resistance of *Arabidopsis* flowers. In conclusion, *10B* is an m^6^A RNA demethylase acting in the floral tissue and contributes to both variable gene expression and heat responsiveness, all of which is required for reproductive success under environmentally challenging conditions.

## Conclusions

It has been previously suggested that stochastic gene expression is advantageous to unicellular organisms with respect to survival under threatening environmental conditions [11, 15, 20, 23]. Such behavior of variable gene expression was also observed in higher eukaryotes including mammals and was found to be associated with disease susceptibility [13, 39, 40]. Gene expression variability can be determined at different levels from cellular to population scales. Recently, Cortijo et al. investigated inter-individual gene expression variability of *Arabidopsis* during the diurnal circadian oscillation and discovered many HVGs involved in environmental responses [23]. In this study, we showed that *Arabidopsis* flowers exhibit reduced variability of gene expression, which limits the activation of heat-responsive genes to a level below certain critical point and consequently compromises fertility under heat stress (Fig. 2g). Therefore, our work suggests a novel framework that gene expression variability is an important factor impacting heat resilience of *Arabidopsis* and m^6^A RNA methylation is key to gene expression variability.

## Materials and methods

### Plant materials and growth condition

*Arabidopsis* Col-0 and *atalkbh10b-1* (SALK_004215C) mutant plants were grown at 22 °C under 16 h light/8 h dark day/night cycle. Heat stress treatment was performed to 5-week-old plants by elevating the growth temperature to 37 °C for 3 h. Rosette leaves (*n* = 6) and stage 1 to 12 floral buds (*n* = 12) were collected for both RNA-seq (3 biological replicates) and m^6^A-RIP-seq (2 biological replicates).

### NGS library construction

Total RNA was isolated using the Trizol reagent (Invitrogen). For m^6^A-RIP-seq, 50 μg of total RNA was used. Briefly, poly(A)-RNA was selected using the oligo-d(T)25 magnetic beads (Thermo Fisher) and was fragmented using Magnesium RNA Fragmentation Module (NEB) at 86 °C for 7 min. Cleaved RNA fragments were then incubated for 2 h at 4 °C with m^6^A-specific antibody (No. 202003, Synaptic Systems) in the IP buffer (50 mM Tris-HCl, 750 mM NaCl and 0.5% Igepal CA-630). Library preparation was performed using the NEBNext Ultra Directional RNA Library Prep Kit (NEB) following the manufacturer’s instructions. Finally, 150-bp paired-end sequencing (PE150) was carried out on an Illumina Novaseq 6000. For RNA-seq, 3 μg of total RNA was used, and the libraries were constructed by following the same method as described above.

### NGS data analyses

Raw sequencing reads were cleaned using Trimmomatic (version 0.39) to remove reads containing adapter and low-quality sequences [41]. Trimmed reads were then aligned to the *Arabidopsis* reference genome (TAIR10) with default settings using Hisat2 (version 2.2.1) [42]. FPKM values were calculated by StringTie (version 2.1.7) [43]. Visualization of the sequencing data was performed using the Integrative Genomics Viewer (IGV) [44]. For gene expression variability analysis, we used the squared coefficient of variation obtained from the FPKM values of three biological replicates. Coefficient of variation is the ratio of standard deviation to mean. For m^6^A peak calling, MACS2 (version 2.2.7.1) was run with the following parameters: --nomodel,--extsize 50, -p 5e-2, and -g 65084214 [45]. The -g option accounts for the size of the *Arabidopsis* transcriptome. m^6^A peaks identified in both two replicates were annotated by CHIPseeker [46]. Distribution of m^6^A peaks along mRNAs were analyzed by adopting the MeRIP-PF and Guitar scripts [47, 48]. Consensus sequence motif was analyzed using the top 1000 most significant m^6^A-enriched regions. RRACH sequence motif was searched in the selected regions, and its frequency was calculated and visualized using the weblogo tool [49]. The NGS datasets generated in this study is summarized in Additional file 5: Table S4.

### m^6^A-RIP-qPCR

m^6^A-RIP-qPCR experiment was performed as described previously with minor modifications [50] and the Magna RIP™ RNA-Binding Protein Immunoprecipitation Kit (Merck) was used following the manufacturer’s instruction. Briefly, 300 μg of total RNA was fragmented using RNA fragmentation buffer [for 1 mL 10X reagents: 800 μL 1 M Tris-HCl (pH 7.0), 100 μL 1 M ZnCl_2_, 100 μL RNase-free H_2_O] to a size of about 250 nucleotides. Fragmented RNA was then precipitated using 2.5 volume of ethanol, 1/10 volume of 3 M NaOH, and 100 μg/mL glycogen at − 80 °C overnight. Precipitated RNA was resuspended in 55 μL RNase-free H_2_O, 5 μL of which was kept as input sample. The remaining RNA was incubated with 5 μg of m^6^A-specific antibody (No. 202003, Synaptic Systems) overnight at 4 °C. Immunoprecipitated RNA was isolated using magnetic beads and the beads were washed five times with 500 μL of cold RIP wash buffer. After resuspending the beads in 150 μL of proteinase K buffer (117 μL of RIP wash buffer, 15 μL of 10% SDS, 18 μL of 10 mg/mL proteinase K) and incubation at 55 °C for 30 min, both input and immunoprecipitated RNA was isolated by the phenol:chloroform method and resuspended in 20 μL of RNase-free H_2_O. Reverse transcription reaction was performed using the ReverTra Ace qPCR RT Master Mix (Toyobo). The resulting first-strand cDNA was diluted four-fold with DEPC-treated water and 1.5 μL was used for a 20-μL qPCR reaction. Real-time qPCR was carried out using ChamQ Universal SYBR qPCR Master Mix (Vazyme) in the CFX96 Connect Real-time PCR Detection system (BioRad). *Actin2* was used as an internal control and the sequences of the primers are provided in Additional file 6: Table S5.

### Heat resistance phenotyping

Heat tolerance test at reproductive stage was carried out as described previously [4]. Briefly, plants were grown under normal growth condition set at 22 °C and 16 h light/8 h dark day/night cycle. Heat stress of 37 °C was treated for 6 h when the first flower opened. After the heat treatment, plants were moved to normal growth condition and continued to grow for 10 days. Silique length was measured from eight individual plants per genotype.

## Supplementary Information

Additional file 1: Fig. S1. Divergent transcriptomic changes of leaves and flowers in heat. Fig. S2. Comparison of m^6^A enrichment in two biological replications. Fig. S3. Detected sequence motifs of m^6^A-modified sites. Fig. S4. m^6^A peaks detected in different clusters. Fig. S5. Correlation between m^6^A and expression levels. Fig. S6. Correlation between FC m^6^A and FC expression. Fig. S7. Increased m^6^A RNA modification in *Arabidopsis* flowers. Fig. S8. Overlap of lowly variable genes identified from different datasets. Fig. S9. Inverse correlation of m^6^A levels and gene expression variability. Fig. S10. Lowly variable genes show stronger m^6^A RNA modification. Fig. S11. Enrichment of genes associated with low expression variability. Fig. S12. Expression profiling of genes encoding RNA demethylases. Fig. S13. An exemplary locus for increased m^6^A in *10b-1*. Fig. S14. Density distribution of m^6^A enrichment in *Arabidopsis* leaves. Fig. S15. Exemplary loci with decreased expression variability in *10b-1*. Fig. S16. Stress-granule association of lowly variable transcripts.

Additional file 2: Table S1. LVGs identified by different datasets.

Additional file 3: Table S2. Expression variability and m^6^A peak numbers.

Additional file 4: Table S3. Hypermethylated genes in leaves and flowers of *10b-1*.

Additional file 5: Table S4. Summary of sequencing data generated in this study.

Additional file 6: Table S5. Sequences of oligonucleotides.

Additional file 7. Review history.

## Data Availability

The datasets supporting the conclusions of this article are available in the SRA repository, PRJNA793364 (https://www.ncbi.nlm.nih.gov/bioproject/PRJNA793364/) [51] and summarized in Additional file 5: Table S4. The analyses were performed using the standard codes instructed by the tools described in the “Methods” section, and the custom codes used in this study are available under MIT license at GitHub (https://github.com/JungnamChoLab/RNA_Methylation) and Zenodo (10.5281/zenodo.7302465) [52].
