# Supplementary material for: m6A RNA methylation impairs gene expression variability and reproductive thermotolerance in Arabidopsis
Source: Genome Biol. 2022 Nov 23;23:244. doi: 10.1186/s13059-022-02814-8 (PMC9686071; doi:10.1186/s13059-022-02814-8)
Supplement: Supplementary file 1 — Additional file 1: Fig. S1. Divergent transcriptomic changes of leaves and flowers in heat. Fig. S2. Comparison of m6A enrichment in two biological replications. Fig. S3. Detected sequence motifs of m6A-modified sites. Fig. S4. m6A peaks detected in different clusters. Fig. S5. Correlation between m6A and expression levels. Fig. S6. Correlation between FC m6A and FC expression. Fig. S7. Increased m6A RNA modification in Arabidopsis flowers. Fig. S8. Overlap of lowly variable genes identified from different datasets. Fig. S9. Inverse correlation of m6A levels and gene expression variability. Fig. S10. Lowly variable genes show stronger m6A RNA modification. Fig. S11. Enrichment of genes associated with low expression variability. Fig. S12. Expression profiling of genes encoding RNA demethylases. Fig. S13. An exemplary locus for increased m6A in 10b-1. Fig. S14. Density distribution of m6A enrichment in Arabidopsis leaves. Fig. S15. Exemplary loci with decreased expression variability in 10b-1. Fig. S16. Stress-granule association of lowly variable transcripts. [file 13059_2022_2814_MOESM1_ESM.pdf]

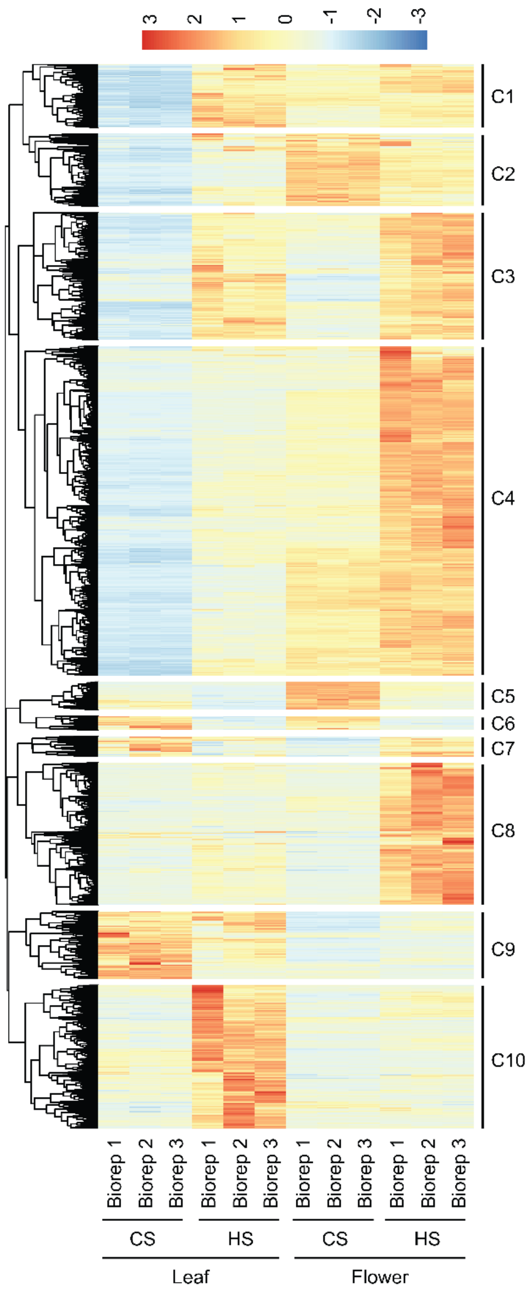

Fig. S1 | Divergent transcriptomic changes of leaves and flowers in heat.

Heatmap of gene expression levels in the control and heat-stressed wt *Arabidopsis* leaves and flowers. Each row represents individual gene and genes are grouped to ten clusters by their expression pattern. Genes with FPKM of at least 1 in any of the samples were only considered. CS, control sample; HS, heat-stressed sample; Biorep, biological replication; C, cluster.

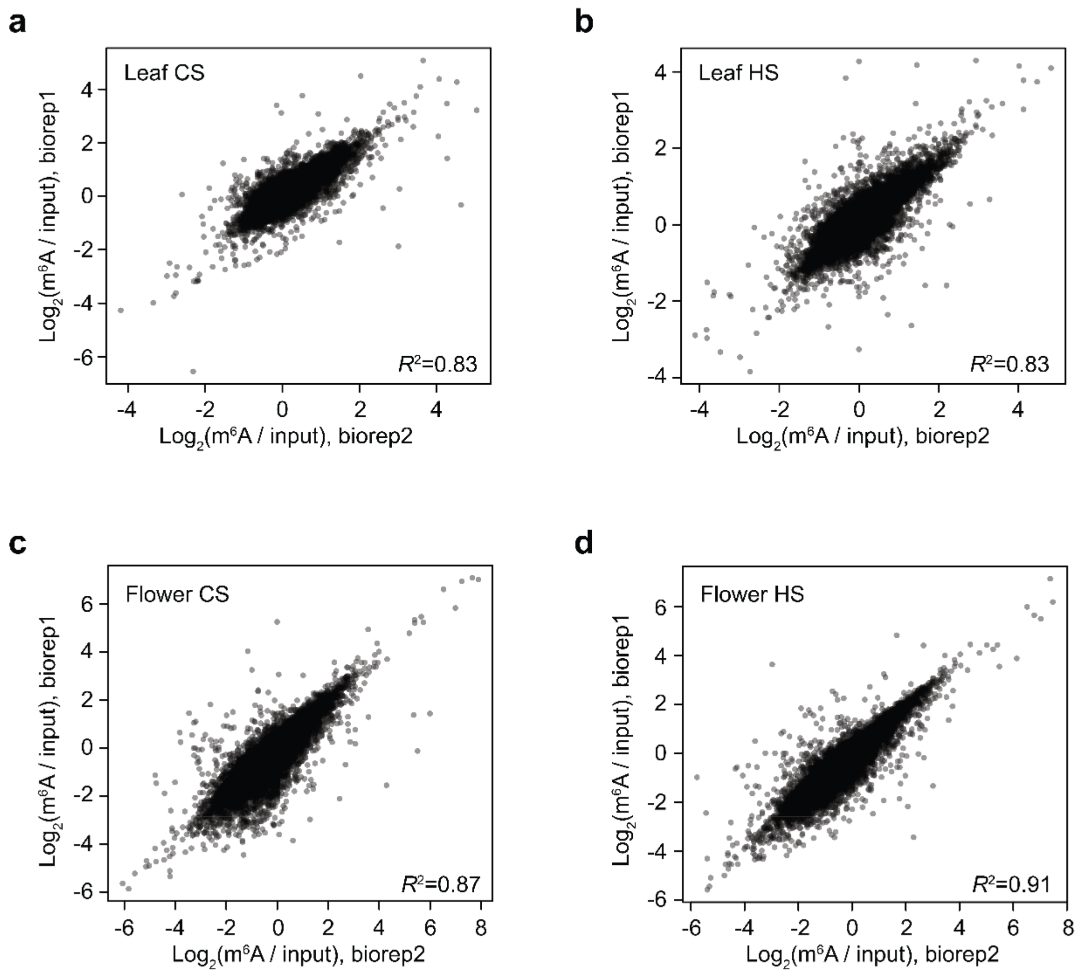

Fig. S2 | Comparison of m^{6}A enrichment in two biological replications.

a-**d**. Enrichment of m^{6}A determined by log$_2$-fold change of m^{6}A-immunoprecipitated to input RNA levels of non-stressed leaves (**a**), heat-stressed leaves (**b**), non-stressed flowers (**c**), and heat-stressed flowers (**d**). CS, control sample; HS, heat-stressed sample; biorep, biological replication. Pearson's product-moment correlation was used for statistical analyses.

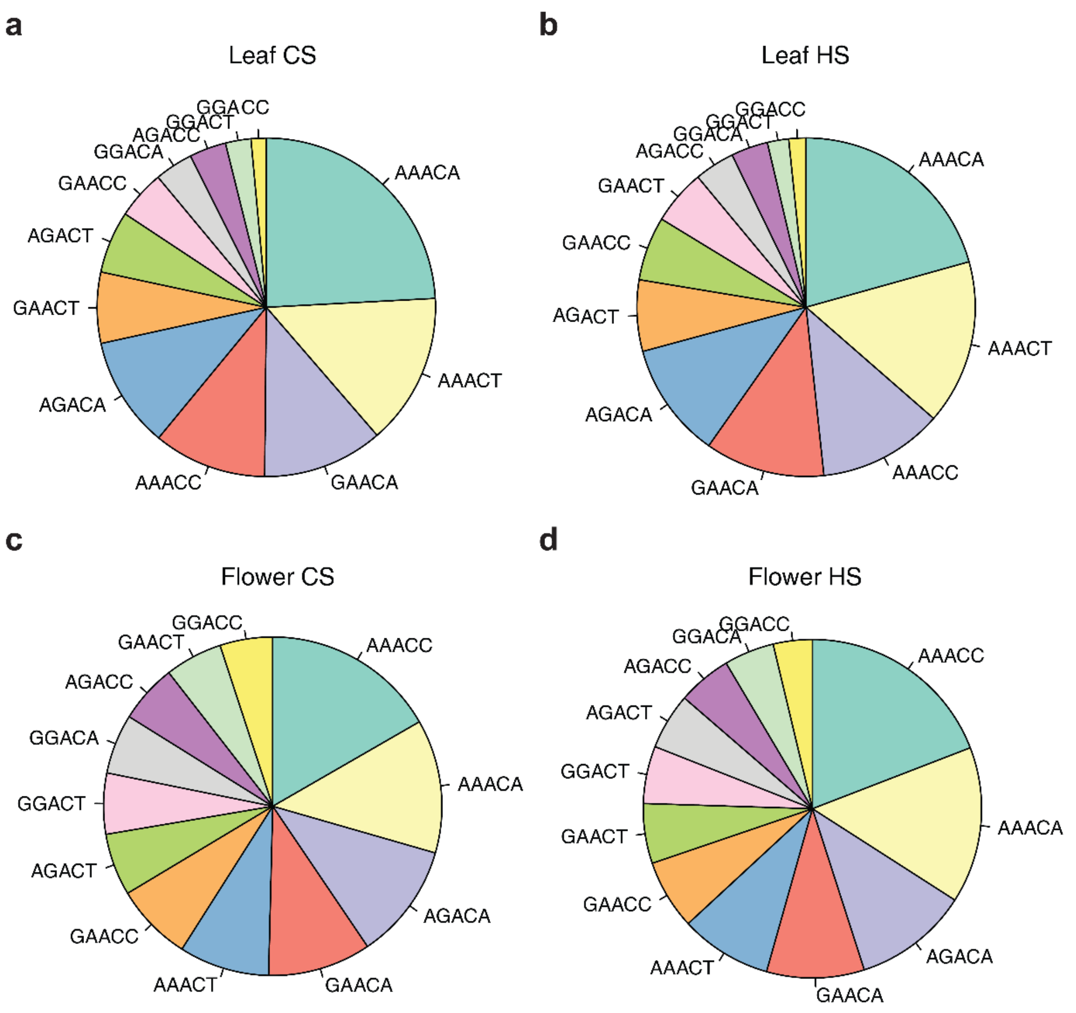

Fig. S3 | Detected sequence motifs of m^{6}A-modified sites.

a-d. Fraction of sequence motifs detected in m^{6}A-modified sites identified from non-stressed leaves (**a**), heat-stressed leaves (**b**), non-stressed flowers (**c**), and heat-stressed flowers (**d**). CS, control sample; HS, heat-stressed sample.

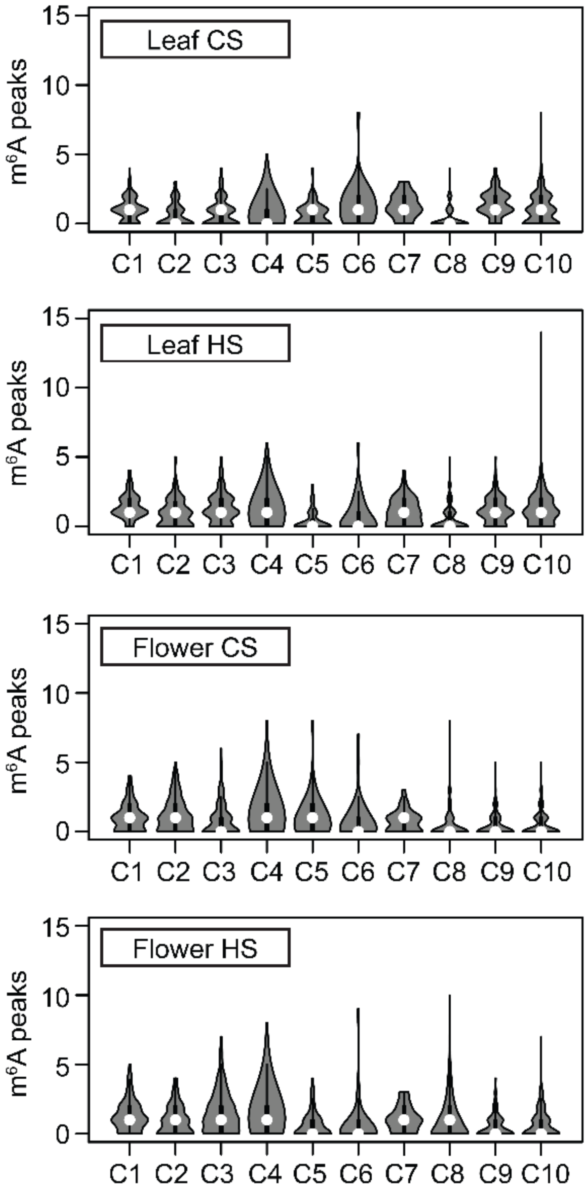

Fig. S4 | m^{6}A peaks detected in different clusters.

Violin plots displaying the distribution of the number of m^{6}A peaks per transcript in different samples. CS, control sample; HS, heat-stressed sample; open circles, median levels; black rectangles, interquartile range.

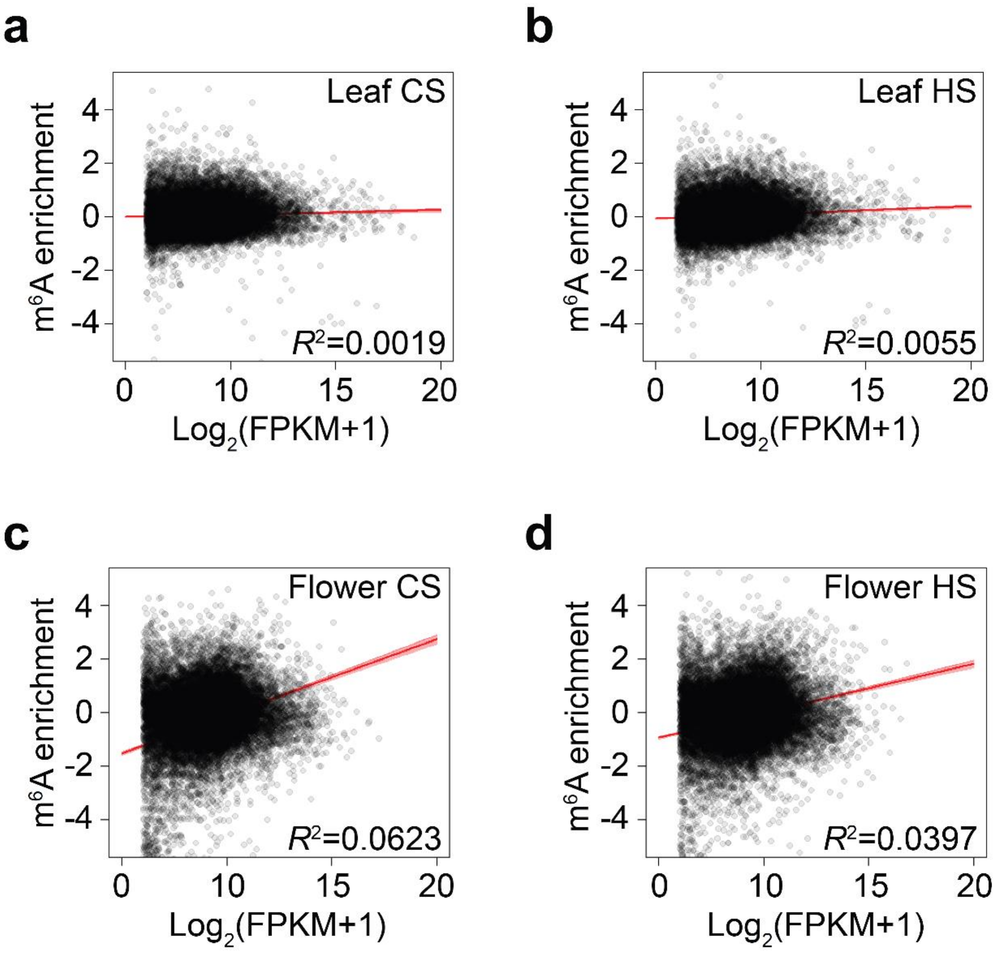

Fig. S5 | Correlation between m^{6}A and expression levels.

a-d. Scatter plots for m^{6}A enrichment and log$_2$-converted FPKM values for non-stressed leaves (**a**), heat-stressed leaves (**b**), non-stressed flowers (**c**), and heat-stressed flowers (**d**). CS, control sample; HS, heat-stressed sample. Pearson's product-moment correlation was used for statistical analyses.

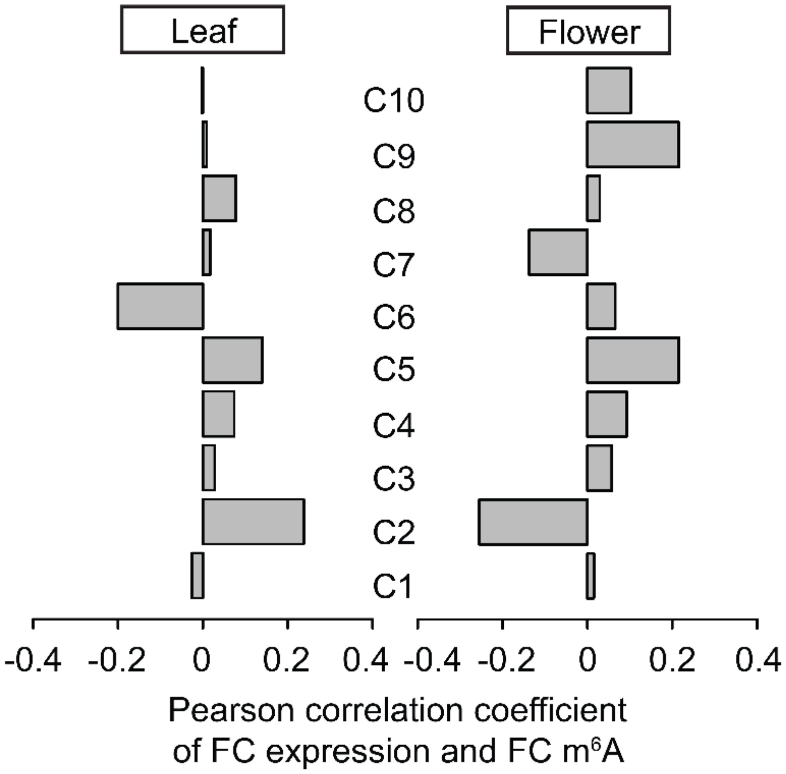

Fig. S6 | Correlation between FC m^{6}A and FC expression.

Pearson correlation coefficient of fold change of expression and m^{6}A enrichment in heat. m^{6}A enrichment was determined by the log2-fold change of m^{6}A levels to input levels. Gene clusters are as defined in Fig. S1.

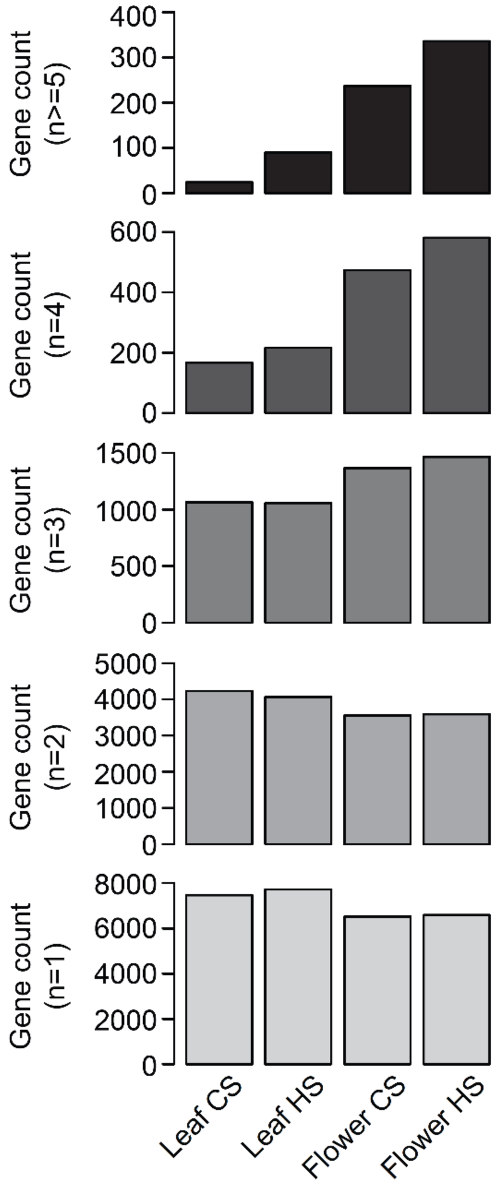

Fig. S7 | Increased m^{6}A RNA modification in *Arabidopsis* flowers.

Count of genes that contain the m^{6}A peaks for the indicated numbers (from bottom to top, 1 to 5). CS, control sample; HS, heat-stressed sample. Raw data is provided in Table S3.

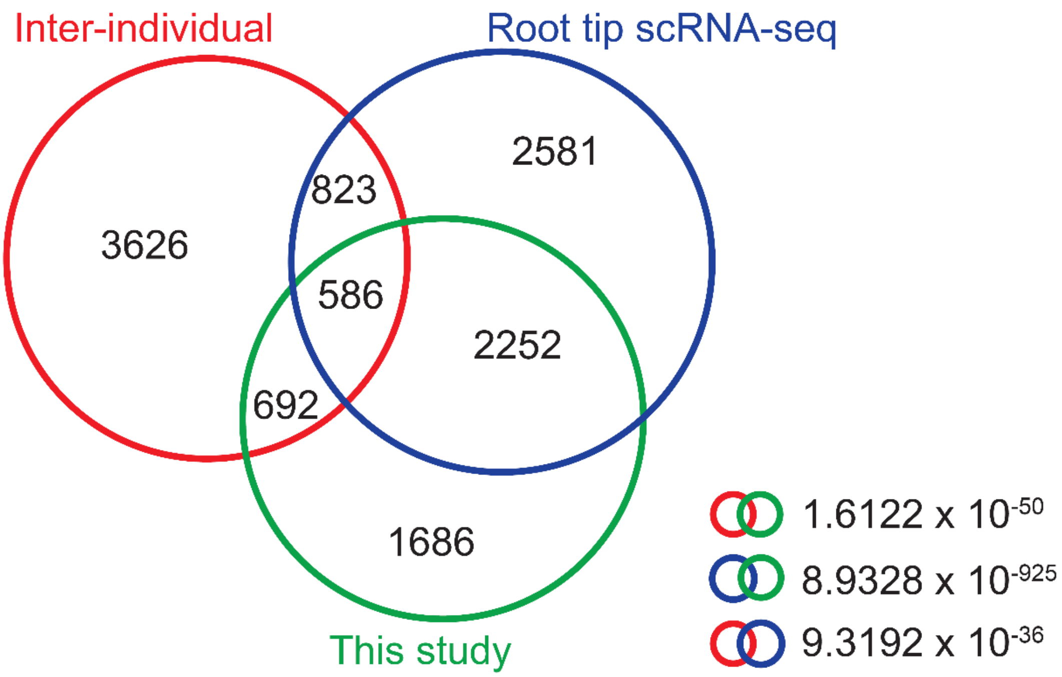

Fig. S8 | Overlap of lowly variable genes identified from different datasets.

Overlap of lowly variable genes (LVGs) determined from various datasets. Inter-individual LVGS are as identified in the study of Cortijo et al (doi: 10.15252/msb.20188591). Root tip scRNA-seq data was obtained from the work of Zhang et al (doi: 10.1016/j.molp.2019.04.004). LVGs are as defined in Fig. 1e and those found in the non-stressed wt flower samples were used. *P* values were obtained by hypergeometric test and are shown for each combination of datasets. The full list of LVGs used in this analysis is provided in Table S4.

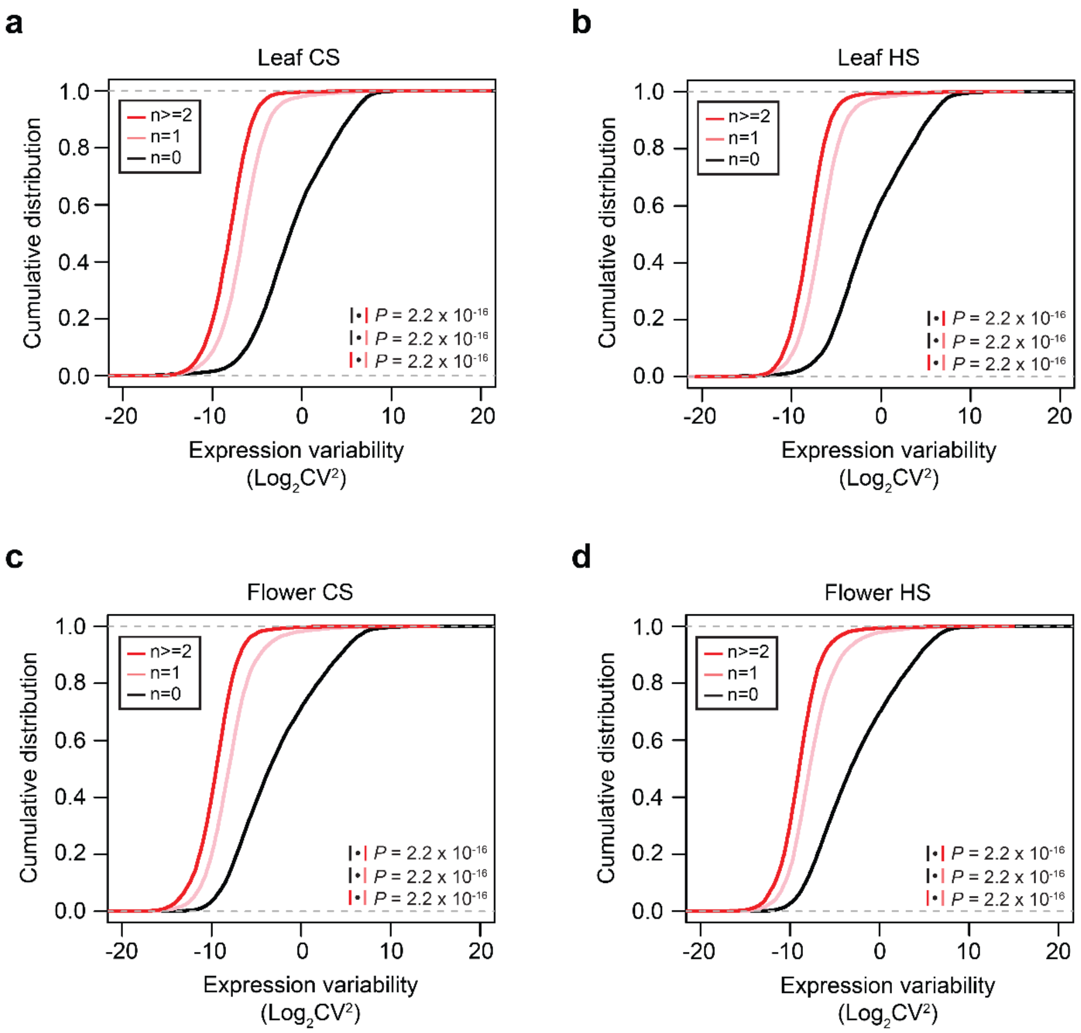

Fig. S9 | Inverse correlation of m^{6}A levels and gene expression variability.

a-d. Cumulative distribution of gene expression variability in non-stressed leaves (**a**), heat-stressed leaves (**b**), non-stressed flowers (**c**) and heat-stressed flower (**d**) for transcripts containing different number of m^{6}A peaks. Gene expression variability was defined as the log2-converted coefficient of variation (CV^2) obtained from three biological replications of RNA-seq data. CS, control sample; HS, heat-stressed sample. Raw data is provided in Table S3.

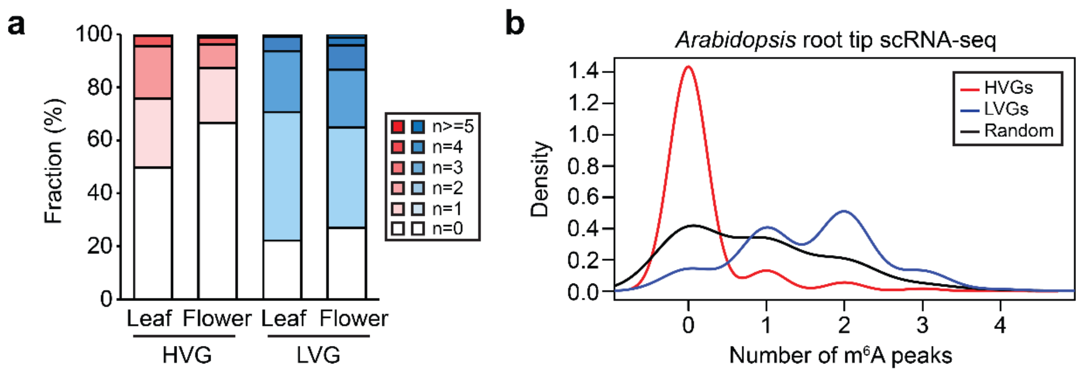

Fig. S10 | Lowly variable genes show stronger m^{6}A RNA modification.

a. Fraction of genes with indicated number of m^{6}A peaks. HVGs and LVGs were obtained from the study of Cortijo et al (doi: 10.15252/msb.20188591). **b**. Density distribution of m^{6}A peak number per transcript. HVGs and LVGs were identified from single-cell RNA-seq data generated from *Arabidopsis* root tip (doi: 10.1016/j.molp.2019.04.004).

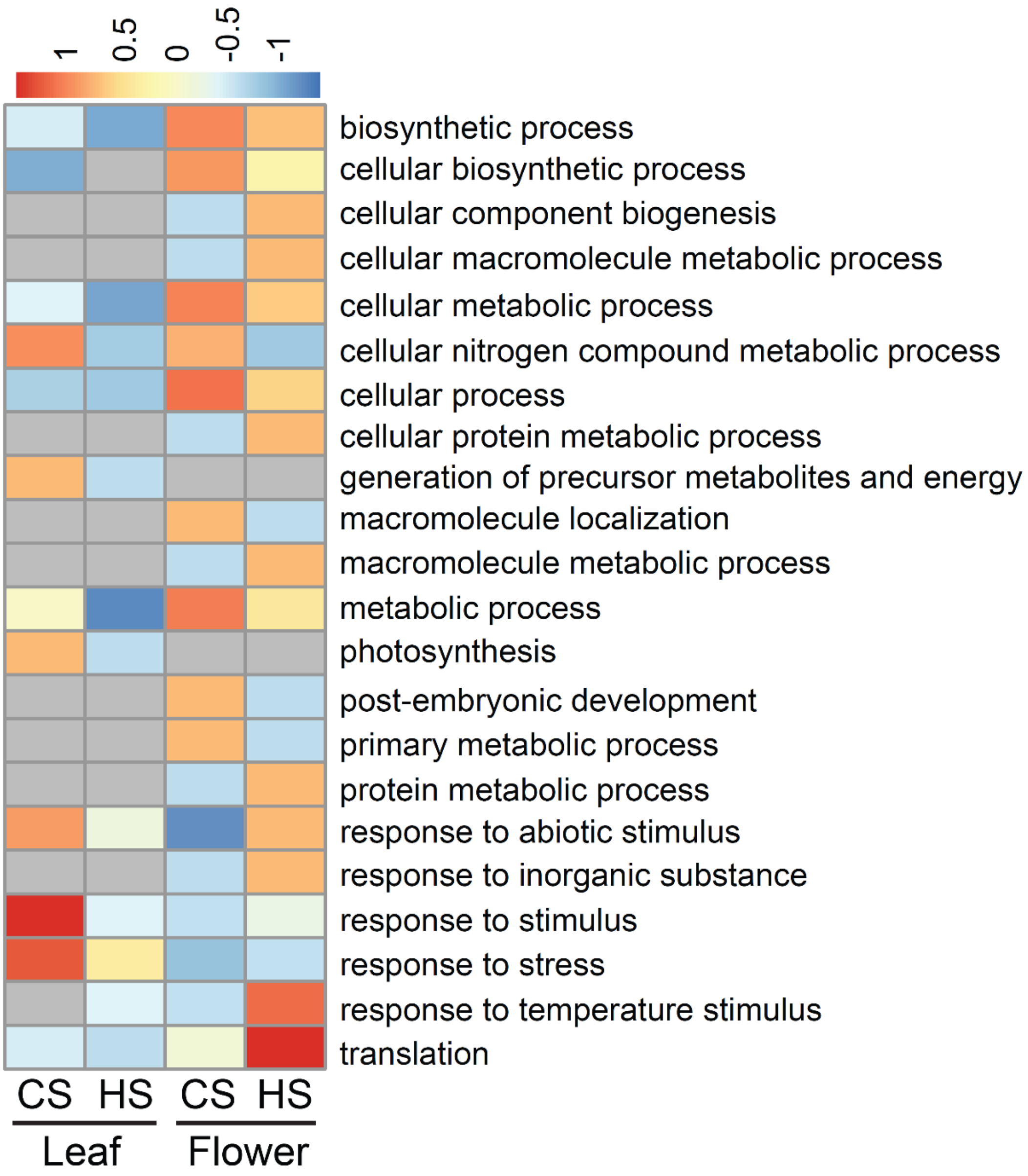

Fig. S11 | Enrichment of genes associated with low expression variability.

GO analyses of LVGs identified from each sample indicated. LVGs are as defined in Fig. 1e. CS, control sample; HS, heat-stressed sample.

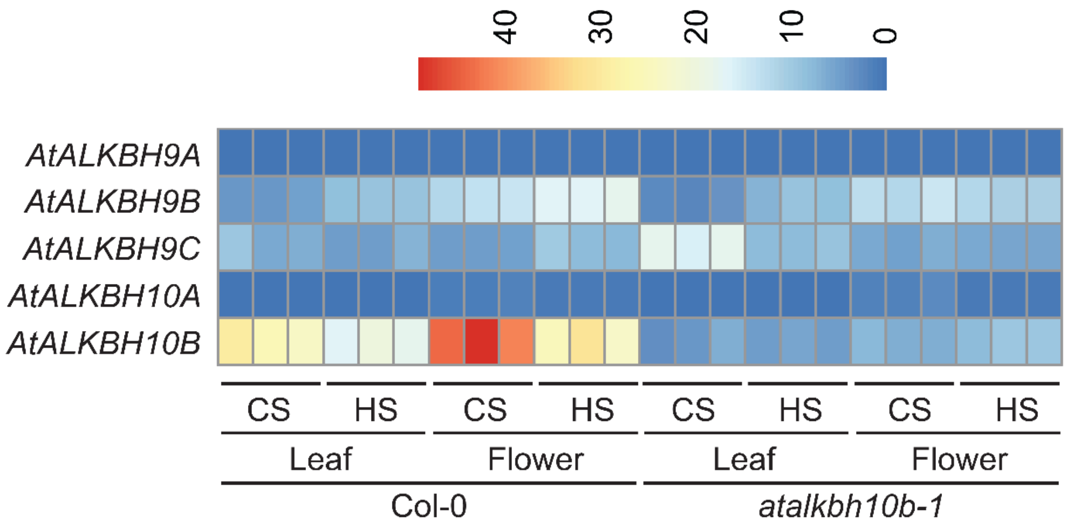

Fig. S12 | Expression profiling of genes encoding RNA demethylases.

Heatmap of gene expression profile of five RNA demethylases in *Arabidopsis*. CS, control sample; HS, heat-stressed sample.

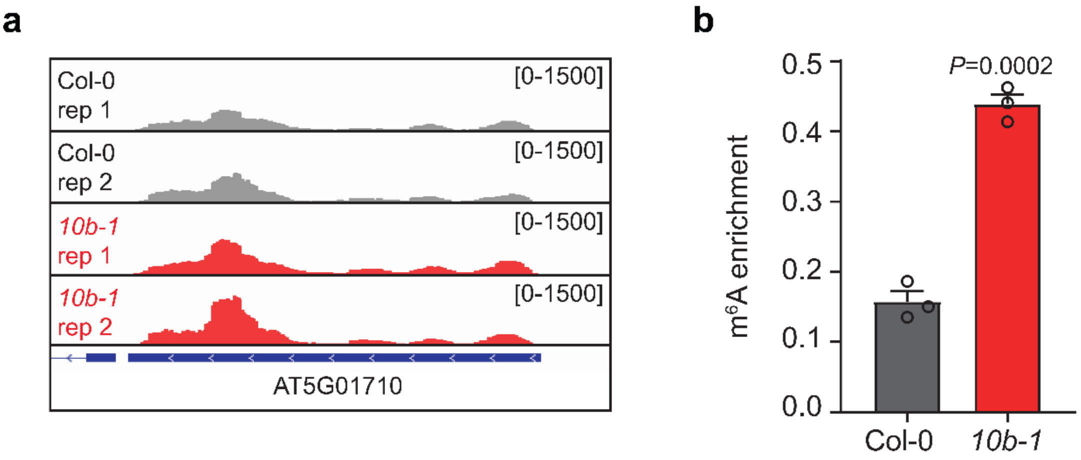

Fig. S13 | An exemplary locus for increased m^{6}A in *10b-1*.

a, A genome browser snapshot showing the coverage of m^{6}A-immunoprecipitated RNA of the wt and *10b-1* leaves. **b**, A qPCR validation of the m^{6}A levels for the locus shown in **a**. Rep, biological replication. Values are mean ± s.d. from three biological replications. *P* values were obtained by the two-tailed Student's t-test.

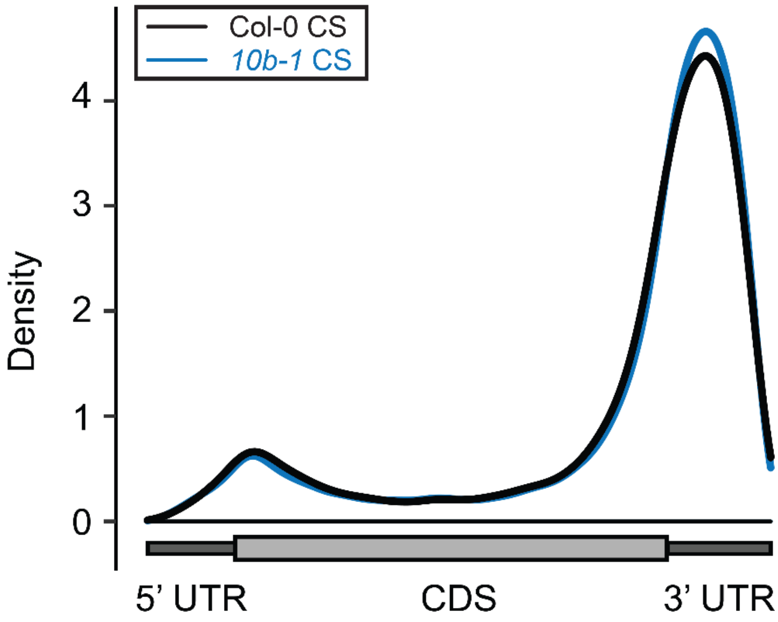

Fig. S14 | Density distribution of m^{6}A enrichment in *Arabidopsis* leaves.

Distribution of m^{6}A in wt and *10b-1* leaves. UTR and CDS are marked as dark and light grey boxes, respectively. CS, non-stressed control condition.

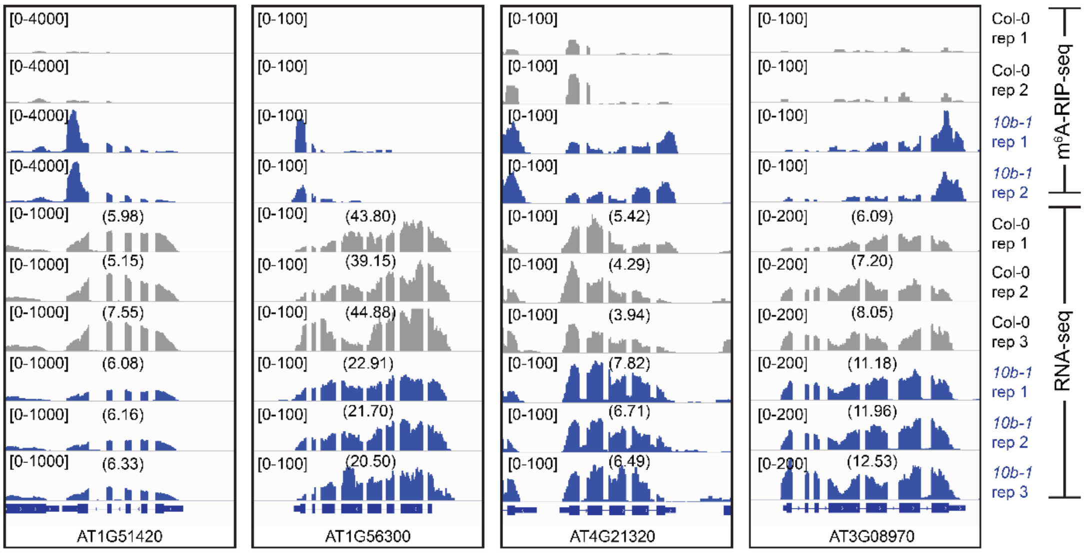

Fig. S15 | Exemplary loci with decreased expression variability in *10b-1*.

Genome browser snapshots showing m^{6}A-RIP-seq and RNA-seq generated from the flowers of wt and *10b-1*. AT1G56300 encodes a DnaJ-domain protein that interacts with heat shock proteins. AT4G21320 encodes HEAT-STRESS-ASSOCIATED 32. AT3G08970 encodes THERMOSENSITIVE MALE STERILE 1. Rep, biological replication. Numbers in parentheses indicate FPKM of corresponding samples.

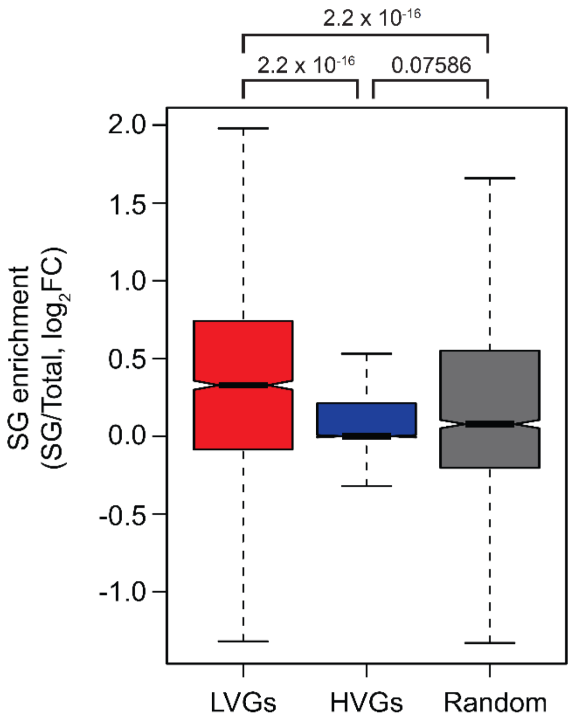

Fig. S16 | Stress-granule association of lowly variable transcripts.

SG enrichment levels of LVGs and HVGs in comparison with randomly selected genes. LVGs and HVGs are the top 2,000 transcripts with the lowest and highest gene expression variability in the non-stressed wt flower samples. SG enrichment data was obtained from the study of Kim et al (doi: 10.1038/s41477-021-00867-4). *P* values were obtained by the one-sided Wilcoxon rank sum test.
