# Supplementary material for: m6A RNA methylation impairs gene expression variability and reproductive thermotolerance in Arabidopsis
Source: Genome Biol. 2022 Nov 23;23:244. doi: 10.1186/s13059-022-02814-8 (PMC9686071; doi:10.1186/s13059-022-02814-8)
Supplement: Supplementary file 7 — Additional file 7. Review history. [file 13059_2022_2814_MOESM7_ESM.docx]

Review History

**First round of review**

**Reviewer 1**

**Were you able to assess all statistics in the manuscript, including the appropriateness of statistical tests used?**

No, I do not feel adequately qualified to assess the statistics.

**Comments to author:**

The authors employed MeRIP-seq and RNA-seq for transcripts in Arabidopsis under normal and heat conditions and proposed that m6A modification mediates avoidance strategies in plants under heat stress. All the experimental results are obtained based on the sequencing results without any experimental verification. However, MeRIP-seq has only two biological replicates. As can be seen from Figure 3e, the repeatability of the two biological replicates in flowers is not ideal. Therefore, I am concerned about the accuracy of the authors' conclusions. And also, the relationship between ALKBH10B and Arabidopsis floral development has been reported in 2017. Therefore, I am also worried about the innovation of this research. There is a confusing use of m6A peak and m6A level, which is not rigorous. The author also has major problems in the paper writing, and many descriptions throughout the article are incomprehensible. The main concerns are listed in the following:

1. Figure 1a indicates the divergent response to heat stress in leaf and flower tissues. The author indicates some clusters showed different expression patterns between leaves and flowers but does not give specific functions of these clusters. Also, some clusters are also ignored, for example, cluster 2 is upregulated in leaves but downregulated in flowers after heat treatment.

2. Are the differentially expressed genes in figure 1a all modified by m6A modification in figure 1b? Although m6A modification is the most common modification in RNA modification, current reports indicate that differentially expressed genes with m6A modification account for a small proportion of the transcriptome data, and the author should indicate the proportion here.

3. Line 115-118: The authors point out the changes in m6A peak numbers in clusters 2, 3, 4, and 8. what is the actual biological significance of the changes in m6A peak numbers. The effect of m6A on gene expression should depend on the m6A level change of the gene, not the number of peaks.

4. The author has addressed that m6A enrichment showed a weak correlation with expression levels Line 119-122. Why did the author focus on the relationship between m6A and expression levels again in Line 125-131?

5. The gene numbers in Figure 2b and 2c appear to be contradictory. For example, in Figure 2b, under normal conditions, there are about 3000 genes in leaves, but in 2c, the number of transcripts containing only one m6A peak in leaves reaches about 7000.

6. Line 154-158: the author stated gene expression variability in this study is similar to sc transcriptome data. It's not clear what does similarity refer to, the number of genes?

7. Line 174-175 unclear writing: "Consistently, LVGs mostly contained at least one m6A peak, whereas HVGs 174 exhibited very weak RNA methylation (Fig. 3g)." The first half of the sentence describes the m6A peak, and the second half describes the m6A level. These two descriptions are not comparable.

8. Line 145-150: Why do the authors still point out that m6A has no correlation with expression level previously, and then concluded that m6A showed a negative correlation with expression level?

9. Line 202-204: The authors point to a comparison of expression level changes in leaves, but describe trends in expression level changes in flowers. Why?

10. The cumulative distribution of gene expression in leaves and flowers under normal conditions was analyzed in Figure 3, without the cumulative distribution under heat treatment.

**Reviewer 2**

**Were you able to assess all statistics in the manuscript, including the appropriateness of statistical tests used?**

Yes, and I have assessed the statistics in my report.

**Comments to author:**

The authors aimed to establish a correlation between m6A RNA methylation and gene expression variability by analyzing m6A levels and gene expression levels in the leaf and flower in Arabidopsis under heat stress. Although this work provides some interesting aspects of m6A modification in gene expression variability, the data shown in the manuscript are too preliminary to correlate m6A methylation with gene expression variability. Many issues should be considered and addressed to claim the conclusions.

1. One of the main conclusions in this manuscript is that higher m6A levels is correlated with lower gene expression variability. For this, Figure 3a shows cumulative distribution of gene expression variability in leaf and flower for transcripts containing different number of m6A peaks. Where are the data showing the list of genes containing different number of m6A peaks? Can MeRIP-seq correctly give us the number of m6A marks in each transcript? To confirm the reliability of the MeRIP-seq data, m6A-IP-qPCR should be done for selected genes containing different number of m6A marks.

2. Figure 1; It is described that gene clusters 1-10 are grouped based on expression pattern similarity, which is hard to understand. What is the meaning of expression pattern similarity? Line 102-106; it is described that Genes in cluster 1 and for instance, were strongly activated upon heat treatment in leaves, while in flowers the level of heat activation was drastically reduced (Fig. 1a). On the other hand, genes in cluster 4 and 9 were hyper-activated under heat stress in flowers (Fig. 1a). These description does not match with the data; the levels of heat activation in cluster 1 and 10 were not reduced in flowers, and the genes in cluster 9 were not hyper-activated under heat stress in flowers.

3. Figure 2 and line 136-138; it is described that as shown in Fig. 2a, the distribution of m6A mark in the flower samples was different from that of leaves exhibiting higher RNA methylation along the CDS. However, the lower levels of m6A mark in the 3'UTR in flower were also observed, which was never described in the text.

4. Figure 3; to obtain more clear correlation between m6A levels and gene expression variability, figure 3a and 3b should be shown for leaf and flowers under heat stress. In line 159-160, it is described that expression variability of the transcripts containing varying number of m6A peaks and found a strong negative correlation (Fig. 3a and b). This description is not clear enough. Because m6A levels increase in flowers, cumulative distribution of gene expression variability in leaf and flower for transcripts containing different number of m6A peaks should be different, which should be described in detail.

5. Figure 3e and 4e; the numbers of FPKM of corresponding genes are shown as an indicator of gene expression variability, which is hard to understand. How are these number correlated with gene expression variability? Why this two genes (At3g18390 and At1g51420) are selected? Are they involved in heat tolerance in flowers? In addition, Genome browser snapshot of only one gene was shown, which is not enough to generalize the correlation between increased m6A level and decreased expression variability. Moreover, to support the MeRIP-seq data, m6A-IP-qPCR should be done for this selected genes.

6. Figure 4 and line 185-189; it is described that the flower samples of 10b-1 exhibited distinct m6A distribution as compared with wt, showing stronger peak around the stop codon (Fig. 4b). This altogether suggests that 10B mediates RNA demethylation mostly in flowers of Arabidopsis. These statements is misleading. Figure 4b also shows that m6A levels in CDS decrease in the mutant, which indicates that 10B increases m6A levels in flowers.

7. Figure 5 line 206-207; the number of aberrant siliques was used to indicate the heat-sensitive phenotypes of the 10b-1 mutant, which is not enough. The pictures showing aberrant siliques and other physiological damages of the mutant should be provided. Although Figure 5c shows an increased m6A level and decreased gene expression variability in alkbh10b mutant, the direct correlation between m6A level and gene expression variability cannot be justified simply based on this result.

8. The methods were poorly described, which is hard to follow many parts of the manuscript. For instance, in line 150-152, it is described that gene expression variability refers to stochastic and noisy expression of genetically identical cells and can be determined by the squared coefficient of variation (CV2). For non-experts in this field, it is hard to understand how gene expression variability is determined.

9. Line 121-123; it is described that RNA methylation does not directly influence the mRNA levels. This is in strong agreement with previous studies that suggested a marginal effect of RNA methylation in mRNA steady-state levels. This is misleading, because many reports demonstrated that RNA methylation affects the mRNA levels in many plant species under both normal and stress conditions.

**Reviewer 3**

**Were you able to assess all statistics in the manuscript, including the appropriateness of statistical tests used?**

Yes, and I have assessed the statistics in my report.

**Comments to author:**

The authors of "m6A RNA methylation impairs gene expression variability and reproductive thermotolerance in Arabidopsis" have revealed the correlation of gene expression variability and m6A enrichment level in different tissues of heat-stressed Arabidopsis, opened a novel view of characterizing heat-responsive genetic/epigenetic changes in plants, emphasized the importance of RNA modifications during environmental variations. Moreover, authors report a demethylase of RNA that exclusively affect the m6A level in follower, as well as the correlation of heat-activated genes and AtALKBH10B. The manuscript is nicely constructed with inventive conclusions. However, I still have several concerns regarding the data analysis and interpretations:

Major concerns:

1. Line 102-105, "As shown in Fig. 1a, flower exhibited a distinct pattern of gene expression under heat stress. Genes in cluster 1 and 10, for instance, were strongly activated upon heat treatment in leaves, while in flowers the level of heat activation was drastically reduced (Fig. 1a)". The statement may not be true since some low- or none-expressed genes were included in the analysis. For example, genes in cluster 10 may not expressed in flowers in both CS and HS conditions. It would be nice if the authors to use genes with RPKM > 1 in both tissues for the analysis.

2. Gene expression variability determined from biological replicates may be due to uncontrolled effect during experimental processes. The analysis based on scRNA-seq data cannot rule out this possibility.

3. In Fig 2a, there is a peak in the 3'UTR for leaf data. How to explain this?

4. In Fig 3a/b, higher expression variability (black cures) could be contributed by low-expressed genes since they are more variable in principle. The authors need to exclude low-expressed genes (i.e., avg RPKM < 1) from the analysis.

Minor comments:

1. Fig1 show that in general level of m6A modification was not significantly changed under heat stress yet the gene expression pattern was drastically changed. In Fig1C authors show that very weak correlation between gene expression and m6A enrichment, however since it is a whole genome analysis, have authors consider use only heat-activated genes like what you did in Fig5a, to narrow down the scale and reduce the dilution effect. Since knockout of 10B leads to increase of m6A enrichment in follow specifically, as illustrated in Fig5a, such effect also revealed in transcriptional level—at least for heat-activated genes. I'm curious to know the correlation of those gene and m6A will be more significant or not.

2. 10B mutant apparently not only alert the heat-activated genes in flower but also in leaf (although the m6A level does not change in leaf), does authors has any hypothesis on this phenomenon?

3. Line 130: "… detect any meaningful levels of correlation …" What do you mean "meaningful"?

4. The Methods part is rather short.

**Authors Response**

**Point-by-point responses to the reviewers’ comments:**

Reviewer #1:

The authors employed MeRIP-seq and RNA-seq for transcripts in Arabidopsis under normal and heat conditions and proposed that m6A modification mediates avoidance strategies in plants under heat stress. All the experimental results are obtained based on the sequencing results without any experimental verification. However, MeRIP-seq has only two biological replicates. As can be seen from Figure 3e, the repeatability of the two biological replicates in flowers is not ideal. Therefore, I am concerned about the accuracy of the authors' conclusions. And also, the relationship between ALKBH10B and Arabidopsis floral development has been reported in 2017. Therefore, I am also worried about the innovation of this research. There is a confusing use of m6A peak and m6A level, which is not rigorous. The author also has major problems in the paper writing, and many descriptions throughout the article are incomprehensible. The main concerns are listed in the following:

1. Figure 1a indicates the divergent response to heat stress in leaf and flower tissues. The author indicates some clusters showed different expression patterns between leaves and flowers but does not give specific functions of these clusters. Also, some clusters are also ignored, for example, cluster 2 is upregulated in leaves but downregulated in flowers after heat treatment.

*Reply: Since the comparison of leaves and flowers in their heat-induced transcriptomic changes is not in fact the central part of our work and was documented already in several previous reports, Fig. 1a is moved to Fig. S1 in the revised version. In addition, due to the word count limit of Short Report format, we hope it can be understood that this part had to be written concisely.*

2. Are the differentially expressed genes in figure 1a all modified by m6A modification in figure 1b? Although m6A modification is the most common modification in RNA modification, current reports indicate that differentially expressed genes with m6A modification account for a small proportion of the transcriptome data, and the author should indicate the proportion here.

*Reply: It is true that not all genes displayed in Fig. 1a are m6A-modified. With Fig. 1b (now in Fig. S4), we wanted to show that m6A RNA modification does not change dramatically upon heat stress and in different tissues, while RNA levels are vastly changed. Fig. S5 is also a nice evidence that RNA levels are poorly correlated with m6A enrichment levels. We clarified this point in the revised manuscript (lines 97-103).*

3. Line 115-118: The authors point out the changes in m6A peak numbers in clusters 2, 3, 4, and 8. what is the actual biological significance of the changes in m6A peak numbers. The effect of m6A on gene expression should depend on the m6A level change of the gene, not the number of peaks.

*Reply: We fully agree with reviewer that m6A enrichment is apparently a more appropriate measure for m6A level. In some studies, m6A peak number is also used to express m6A level; however, for the consistency of the manuscript, all the data relating m6A peaks was moved to supplementary information (Fig. S4, S7, S9, and S10).*

4. The author has addressed that m6A enrichment showed a weak correlation with expression levels Line 119-122. Why did the author focus on the relationship between m6A and expression levels again in Line 125-131?

*Reply: Fig. 1c (now in Fig. S6) shows correlation between fold changes of expression and m6A enrichment by heat stress, which is different from the correlation of expression and m6A enrichment shown in Fig. S5.*

5. The gene numbers in Figure 2b and 2c appear to be contradictory. For example, in Figure 2b, under normal conditions, there are about 3000 genes in leaves, but in 2c, the number of transcripts containing only one m6A peak in leaves reaches about 7000.

*Reply: Fig. 2b (now in Fig. 1c) and Fig. 2c (now in Fig. S7) are different because m6A-modified transcripts are differently defined; m6A enrichment was used in 2b and m6A peak number was used in 2c. To avoid misunderstanding, 2c is moved to supplementary information.*

6. Line 154-158: the author stated gene expression variability in this study is similar to sc transcriptome data. It's not clear what does similarity refer to, the number of genes?

*Reply: We admit that it was described imprecisely and we rephrased the sentence as following: “genes with invariabe expression in biological replications significantly overlapped with those invariabile genes identified from single-cell (sc) and single-plant RNA-seq datasets (Fig. S8).” (lines 119-121).*

7. Line 174-175 unclear writing: "Consistently, LVGs mostly contained at least one m6A peak, whereas HVGs 174 exhibited very weak RNA methylation (Fig. 3g)." The first half of the sentence describes the m6A peak, and the second half describes the m6A level. These two descriptions are not comparable.

*Reply: We thank reviewer for pointing this out. The sentence now reads: “LVGs contained more m6A-modified transcripts than HVGs (Fig. S10b).” (lines 136-136).*

8. Line 145-150: Why do the authors still point out that m6A has no correlation with expression level previously, and then concluded that m6A showed a negative correlation with expression level?

*Reply: m6A level does not correlate with expression levels (Fig. S5) or fold change expression (Fig. S6), and shows strong negative correlation with expression variability (Fig. 1f and 2c). Expression variability is different from expression level as it refers to the level of dispersion of expression levels in biological replications. We tried to make it clear by explaining it in the main text (lines 115-117), method (lines 229-231), and figure legend (lines 475-477).*

9. Line 202-204: The authors point to a comparison of expression level changes in leaves, but describe trends in expression level changes in flowers. Why?

*Reply: Our work started from a question of why flowers are more susceptible to heat stress. We selected heat-activated genes in leaves and used them as a control gene set reflecting normal heat response. Flowers and 10b-1 mutant (where m6A levels are elevated) exhibit reduced fold change upon heat stress, indicating compromised heat response and tolerance.*

10. The cumulative distribution of gene expression in leaves and flowers under normal conditions was analyzed in Figure 3, without the cumulative distribution under heat treatment.

*Reply: Cumulative distribution under heat treatment is added in Fig. S9.*

Reviewer #2:

The authors aimed to establish a correlation between m6A RNA methylation and gene expression variability by analyzing m6A levels and gene expression levels in the leaf and flower in Arabidopsis under heat stress. Although this work provides some interesting aspects of m6A modification in gene expression variability, the data shown in the manuscript are too preliminary to correlate m6A methylation with gene expression variability. Many issues should be considered and addressed to claim the conclusions.

1. One of the main conclusions in this manuscript is that higher m6A levels is correlated with lower gene expression variability. For this, Figure 3a shows cumulative distribution of gene expression variability in leaf and flower for transcripts containing different number of m6A peaks. Where are the data showing the list of genes containing different number of m6A peaks? Can MeRIP-seq correctly give us the number of m6A marks in each transcript? To confirm the reliability of the MeRIP-seq data, m6A-IP-qPCR should be done for selected genes containing different number of m6A marks.

*Reply: Firstly, a table of list of genes, expression variability, and m6A peak numbers is provided in Supplementary Table S3. Secondly, m6A-RIP-seq data can be analyzed to retrieve the enriched region (which is expressed as “peak” in our manuscript) and the exact number of m6A sites cannot be obtained. The peak calling method is described in the method (lines 231-236). Thirdly, the reliability of our m6A-RIP-seq can be confirmed by their reproducibility between two independent biological replications (Fig. S2) and the qPCR validation presented in Fig. S13.*

2. Figure 1; It is described that gene clusters 1-10 are grouped based on expression pattern similarity, which is hard to understand. What is the meaning of expression pattern similarity? Line 102-106; it is described that Genes in cluster 1 and for instance, were strongly activated upon heat treatment in leaves, while in flowers the level of heat activation was drastically reduced (Fig. 1a). On the other hand, genes in cluster 4 and 9 were hyper-activated under heat stress in flowers (Fig. 1a). These description does not match with the data; the levels of heat activation in cluster 1 and 10 were not reduced in flowers, and the genes in cluster 9 were not hyper-activated under heat stress in flowers.

*Reply: Firstly, genes are clustered by their expression pattern and the sentence is rephrased in the revised figure legend (now in Fig. S1). Secondly, regarding the description of clusters, we rephrased the sentence to be more precise and now it reads: “Genes in cluster 1 and 10, for instance, were strongly activated upon heat treatment in leaves, while in flowers their expression levels were not altered dramatically” (lines 85-87). Thirdly, cluster 9 should be corrected to 8, but we decided to remove that sentence because of the space constraint.*

3. Figure 2 and line 136-138; it is described that as shown in Fig. 2a, the distribution of m6A mark in the flower samples was different from that of leaves exhibiting higher RNA methylation along the CDS. However, the lower levels of m6A mark in the 3'UTR in flower were also observed, which was never described in the text.

*Reply: We thank reviewer for pointing this out. The description is added in the revised manuscript (lines 108-110).*

4. Figure 3; to obtain more clear correlation between m6A levels and gene expression variability, figure 3a and 3b should be shown for leaf and flowers under heat stress. In line 159-160, it is described that expression variability of the transcripts containing varying number of m6A peaks and found a strong negative correlation (Fig. 3a and b). This description is not clear enough. Because m6A levels increase in flowers, cumulative distribution of gene expression variability in leaf and flower for transcripts containing different number of m6A peaks should be different, which should be described in detail.

*Reply: We added the cumulative plots for the heat-stressed samples in Fig. S9. In fact, the distribution of expression variability for methylated transcripts in the flower sample is shifted to a lower level compared to the leaf sample. This can be most clearly seen around expression variability around -10. In addition, to further support the negative correlation between m6A levels and expression variability, Fig. 1f is also added in the revised manuscript.*

5. Figure 3e and 4e; the numbers of FPKM of corresponding genes are shown as an indicator of gene expression variability, which is hard to understand. How are these number correlated with gene expression variability? Why this two genes (At3g18390 and At1g51420) are selected? Are they involved in heat tolerance in flowers? In addition, Genome browser snapshot of only one gene was shown, which is not enough to generalize the correlation between increased m6A level and decreased expression variability. Moreover, to support the MeRIP-seq data, m6A-IP-qPCR should be done for this selected genes.

*Reply: Firstly, gene expression variability is a measure of divergence of gene expression levels between biological replications and its negative correlation with m6A level can be directly supported by Fig. 1f ad 2c, which we believe is robust enough. Secondly, Fig. 4e (now in Fig. S15) is just one of the examples that shows an increased m6A level is associated with less variable expression levels between replications, and its relevance to heat tolerance is not in fact known. Thirdly, we supplemented Fig. S13 with qPCR validation to show the reproducibility of our sequencing data.*

6. Figure 4 and line 185-189; it is described that the flower samples of 10b-1 exhibited distinct m6A distribution as compared with wt, showing stronger peak around the stop codon (Fig. 4b). This altogether suggests that 10B mediates RNA demethylation mostly in flowers of Arabidopsis. These statements is misleading. Figure 4b also shows that m6A levels in CDS decrease in the mutant, which indicates that 10B increases m6A levels in flowers.

*Reply: Firstly, we detailed the description which now reads: “the flower sample of 10b-1 exhibited distinct m6A distribution as compared with wt, showing stronger peak around the stop codon and weaker enrichment along the CDS (Fig. 2a)” (lines 149-151). Secondly, distribution of m6A shown in Fig. 4b (now in Fig. 2a) does not necessarily reflect m6A level as it only shows the m6A peak occurrence along transcripts. Our conclusion that 10B acts strongly in flowers is supported by Fig. 4c (now in Fig. 2b).*

7. Figure 5 line 206-207; the number of aberrant siliques was used to indicate the heat-sensitive phenotypes of the 10b-1 mutant, which is not enough. The pictures showing aberrant siliques and other physiological damages of the mutant should be provided. Although Figure 5c shows an increased m6A level and decreased gene expression variability in alkbh10b mutant, the direct correlation between m6A level and gene expression variability cannot be justified simply based on this result.

*Reply: Firstly, we added Fig. 2f in the revised manuscript as per reviewer’s suggestion. Secondly, the inverse correlation between m6A level and expression variability is supported by multiple evidence presented in Fig. 1f, 2c, S9, S10, and S15, which we believe can justify our conclusion.*

8. The methods were poorly described, which is hard to follow many parts of the manuscript. For instance, in line 150-152, it is described that gene expression variability refers to stochastic and noisy expression of genetically identical cells and can be determined by the squared coefficient of variation (CV2). For non-experts in this field, it is hard to understand how gene expression variability is determined.

*Reply: We detailed how expression variability is calculated by describing it in the main text (lines 115-117), method (lines 229-231), and figure legend (lines 475-477).*

9. Line 121-123; it is described that RNA methylation does not directly influence the mRNA levels. This is in strong agreement with previous studies that suggested a marginal effect of RNA methylation in mRNA steady-state levels. This is misleading, because many reports demonstrated that RNA methylation affects the mRNA levels in many plant species under both normal and stress conditions.

*Reply: We agree with reviewer that the sentence can be misleading. Many reports mostly demonstrated the roles of RNA methylation on individual or few selected genes, and from that sentence we wanted to suggest that RNA methylation has little impact on transcriptome. The sentence is rephrased as following: “This is in agreement with previous studies that suggested a negligible effect of RNA methylation in transcriptome-wide mRNA steady-state levels in Arabidopsis” (lines 104-105).*

Reviewer #3:

The authors of "m6A RNA methylation impairs gene expression variability and reproductive

thermotolerance in Arabidopsis" have revealed the correlation of gene expression variability and m6A enrichment level in different tissues of heat-stressed Arabidopsis, opened a novel view of characterizing heat-responsive genetic/epigenetic changes in plants, emphasized the importance of RNA modifications during environmental variations. Moreover, authors report a demethylase of RNA that exclusively affect the m6A level in follower, as well as the correlation of heat-activated genes and AtALKBH10B. The manuscript is nicely constructed with inventive conclusions. However, I still have several concerns regarding the data analysis and interpretations:

Major concerns:

1. Line 102-105, "As shown in Fig. 1a, flower exhibited a distinct pattern of gene expression under heat stress. Genes in cluster 1 and 10, for instance, were strongly activated upon heat treatment in leaves, while in flowers the level of heat activation was drastically reduced (Fig. 1a)". The statement may not be true since some low- or none-expressed genes were included in the analysis. For example, genes in cluster 10 may not expressed in flowers in both CS and HS conditions. It would be nice if the authors to use genes with RPKM > 1 in both tissues for the analysis.

*Reply: We are grateful to reviewer for raising this issue. It is true that lowly expressing genes might be underrepresented in the m6A analyses. However, the main conclusions of that part are 1) leaves and flowers respond differently to heat in transcriptome, and 2) transcriptome changes dramatically in heat but m6A does not. We believe that our data is just enough to draw these conclusions and we are in fact concerned that filtering of data points might generate any biases.*

2. Gene expression variability determined from biological replicates may be due to uncontrolled effect during experimental processes. The analysis based on scRNA-seq data cannot rule out this possibility.

*Reply: We admit that variability of gene expression levels can be generated by technical inconsistency. However, if it was caused by technical issues, variability should be observed in all transcripts but our data shows certain level of divergence in variability in different genes. In addition, analysis of expression variability with scRNA-seq data is in fact more reliable because it is calculated from thousands of data points, from which technical bias can be ignored. Most importantly, we observed a remarkable overlap of invariable genes between different datasets (Fig. S8), which indicates that expression variability is not generated simply by technical inconsistency.*

3. In Fig 2a, there is a peak in the 3'UTR for leaf data. How to explain this?

*Reply: We corrected our analysis pipeline and re-generated the density plot.*

4. In Fig 3a/b, higher expression variability (black cures) could be contributed by low-expressed genes since they are more variable in principle. The authors need to exclude low-expressed genes (i.e., avg RPKM < 1) from the analysis.

*Reply: We thank reviewer for pointing out this issue. Low- or non-expressing genes are discarded from the analysis because m6A is less likely detected in lowly expressing genes and coefficient of variation cannot be calculated from non-expressing genes.*

Minor comments:

1. Fig1 show that in general level of m6A modification was not significantly changed under heat stress yet the gene expression pattern was drastically changed. In Fig1C authors show that very weak correlation between gene expression and m6A enrichment, however since it is a whole genome analysis, have authors consider use only heat-activated genes like what you did in Fig5a, to narrow down the scale and reduce the dilution effect. Since knockout of 10B leads to increase of m6A enrichment in follow specifically, as illustrated in Fig5a, such effect also revealed in transcriptional level—at least for heat-activated genes. I'm curious to know the correlation of those gene and m6A will be more significant or not.

*Reply: We agree with reviewer that bulk comparison in transcriptome scale can dilute subtle but important difference. For this reason, we did correlation analyses by clusters and for example, cluster 8 and 10 are good gene sets showing strong heat induction in flowers and leaves, respectively. However, even with those strongly contrasting genes, we were unable to detect any significant correlation to m6A changes.*

2. 10B mutant apparently not only alert the heat-activated genes in flower but also in leaf (although the m6A level does not change in leaf), does authors has any hypothesis on this phenomenon?

*Reply: Our data shows that 10B acts more strongly in flowers but it does not mean that 10B only acts in flowers. As was shown in Fig. S12, 10B is expressed at fairly high level in leaves (not as high as in flowers but still higher than any other RNA demethylases). Therefore, we speculate that 10B might possess some function in leaves as well but in limited extent compared to that in flowers.*

*3. Line 130: "… detect any meaningful levels of correlation …" What do you mean "meaningful"?*

*Reply: The sentence is rephrased to be more precise (lines 102-103).*

4. The Methods part is rather short.

*Reply: We detailed the methods part as per reviewer’s suggestion.*

**Second round of review**

**Reviewer 1**

The authors used MeRIP-seq and RNA-seq to compare the response patterns of leaves and flowers to heat stress. Higher m6A levels and more LVGs were found in flowers than in leaves. Compared to the previous version, the current manuscript describes it more clearly. But the gene expression variability mentioned in this research is a parameter specific to single-cell sequencing analysis, and although the authors pointed out that the RNA-seq data in this study can be used for single-cell analysis, the evidence is still relatively lacking. The authors should provide stronger evidence to justify the use of RNA-seq for single-cell analysis. There are some questions required to be addressed.

1. The authors point to the m6A peak number is also used to express the m6A level, which is difficult to understand. m6A peak number cannot be used to indicate the m6A level, as each peak may be either up-regulated or down-regulated.

2. Line 117-121, the authors elucidated that genes with invariable expression significantly overlapped with LVGs identified from sc and Inter-individual. How does the author conclude that is significant? It is difficult to understand that the authors used different organizations and treatment conditions to overlap. Is it credible?

3. Please author to give more reasonable and more evidence that RNA-seq data in this study can be used for single-cell analysis. Whether there is any literature using a similar approach, otherwise this is highly debated.

4. Line 123, it should be corrected to Fig. 1d and e.

**Reviewer 2**

The authors addressed most of my comments and suggestions, which clarified and improved the manuscript. However, several key points still need to be considered.

1. Line 123; Fig. 2d and e should be Fig. 1d and e.

2. Line 146-147; the description “we found only limited number of transcripts with altered m6A levels in the leaf sample of 10b-1 (Fig. S13)” is misleading. Fig. S13 does not show the number of transcripts with altered m6A levels but just shows an example of a gene with increased m6A in 10b-1. It would be more informative if the list of the genes with altered m6A levels in 10b mutant is shown in a Supplemental table.

3. Fig. S15; it is still hard to understand how the numbers of FPKM of a gene are correlated with gene expression variability. It would be better to briefly explain it in the figure legend. The Genome browser snapshot of only At1g51420 is shown. Why? Is it involved in heat tolerance in flowers? To generalize the correlation between increased m6A level and decreased expression variability in 10b mutant, the Genome browser snapshots of other genes involved in heat tolerance should be shown.

4. Fig. S16; to better support the possibility that LVGs are enriched in SGs, thereby resulting in low gene expression variability, SG enrichment score of HVGs should be shown and compared.

5. Fig. 2g; what does the each bar represent? What is the difference of each bar? It is described that floral tissues exhibit stronger m6A RNA methylation and lower expression variability, and reduced expression variability in flowers diminishes the gene activation to a level below the critical point. What is the critical point? How was this critical point measured? These are poorly described.

**Reviewer 3**

I thank the authors for addressing my previous concerns. However, I don't think the authors provided the Methods details in the revised manuscript. It's still not easy for me to follow how the analyses were done.

1. The authors mentioned the custom codes is provided at https://github.com/JungnamChoLab. I actually did not find the corresponding codes used for this study.

2. Which software was used for the prediction of consensus sequence motif? How the input sequences were generated for the analysis?

**Authors Response**

**Point-by-point responses to the reviewers’ comments:**

Reviewer #1:

The authors used MeRIP-seq and RNA-seq to compare the response patterns of leaves and flowers to heat stress. Higher m6A levels and more LVGs were found in flowers than in leaves. Compared to the previous version, the current manuscript describes it more clearly. But the gene expression variability mentioned in this research is a parameter specific to single-cell sequencing analysis, and although the authors pointed out that the RNA-seq data in this study can be used for single-cell analysis, the evidence is still relatively lacking. The authors should provide stronger evidence to justify the use of RNA-seq for single-cell analysis. There are some questions required to be addressed.

*Reply: We appreciate for this comment. It is true that gene expression variability is a parameter that is being used often in single-cell (sc) RNA sequencing data; however, it was also studied in other scales, for instance, at organismal level and even in replicates of pooled plants, before scRNA-seq became available. In our work, the scRNA-seq data was used to show that there is some level of similarity in lowly variable genes identified by scRNA-seq and RNA-seq, even though they are determined by different experimental methods. We will address this point in more detail in our answer below.*

1. The authors point to the m6A peak number is also used to express the m6A level, which is difficult to understand. m6A peak number cannot be used to indicate the m6A level, as each peak may be either up-regulated or down-regulated.

*Reply: We fully agree with the reviewer that number of m6A peaks cannot be as accurate as m6A enrichment in expressing the level of RNA methylation, although they show positive correlation. In some papers, m6A peak number is used to classify genes for their RNA methylation levels (10.1371/journal.pgen.1009086; 10.1186/s12864-021-07556-8; 10.3390/plants11010103). In this manuscript, for the analyses that compare m6A levels in different tissues, treatment, and genotypes, m6A enrichment score was used, and m6A peak number was only used when classifying genes with strong or weak RNA methylation.*

2. Line 117-121, the authors elucidated that genes with invariable expression significantly overlapped with LVGs identified from sc and Inter-individual. How does the author conclude that is significant? It is difficult to understand that the authors used different organizations and treatment conditions to overlap. Is it credible?

*Reply: Firstly, we performed hypergeometric test to show the significance of the overlaps. P values are added in Fig. S8 and the full list of LVGs is provided in Table S3, which are described in the figure legend (SI lines 57-59).*

*Secondly, in this particular analysis, we wanted to show that deviation of biological replications of RNA-seq data can be used to study gene expression variability, and to support this idea, we compared LVGs defined by other datasets. Although the tissues and developmental stages of samples used in these datasets are different, we observed a significant similarity in their LVGs. In fact, it was previously shown that noisy expression is dictated by certain intrinsic genomic features (10.1126/science.1255301; 10.15252/msb.20188591; 10.1186/s12864-019-6308-7). Therefore, we believe that our argument can be supported by this data.*

3. Please author to give more reasonable and more evidence that RNA-seq data in this study can be used for single-cell analysis. Whether there is any literature using a similar approach, otherwise this is highly debated.

*Reply: Gene expression variability has been studied in various scales such as population, individual, and currently most commonly single cell levels. Until scRNA-seq became available in plant systems, it was investigated usually in single plant or pooled plants. For example, Bhosale et al (10.1105/tpc.113.112268) and Cortijo et al (10.15252/msb.20188591) studied inter-individual expression variability, and Hirao et al (10.1016/j.jtbi.2015.09.017) investigated gene expression noise among replicates of pooled plants. Therefore, expression data derived from RNA-seq have been commonly used in determining gene expression variability. In this study, we wanted to show that a significant portion of lowly variable genes are commonly found in RNA-seq data of pooled plants (this study), single plant, and single cell.*

4. Line 123, it should be corrected to Fig. 1d and e.

*Reply: It is now corrected in the revised version (line 123).*

Reviewer #2:

The authors addressed most of my comments and suggestions, which clarified and improved the manuscript. However, several key points still need to be considered.

1. Line 123; Fig. 2d and e should be Fig. 1d and e.

*Reply: It is now corrected in the revised version (line 123).*

2. Line 146-147; the description “we found only limited number of transcripts with altered m6A levels in the leaf sample of 10b-1 (Fig. S13)” is misleading. Fig. S13 does not show the number of transcripts with altered m6A levels but just shows an example of a gene with increased m6A in 10b-1. It would be more informative if the list of the genes with altered m6A levels in 10b mutant is shown in a Supplemental table.

*Reply: We identified 1,265 and 6,770 hypermethylated transcripts in 10b-1 in leaves and flowers, respectively. The full list of genes is provided in Table S5 and this is also mentioned in the main text (line 147).*

3. Fig. S15; it is still hard to understand how the numbers of FPKM of a gene are correlated with gene expression variability. It would be better to briefly explain it in the figure legend. The Genome browser snapshot of only At1g51420 is shown. Why? Is it involved in heat tolerance in flowers? To generalize the correlation between increased m6A level and decreased expression variability in 10b mutant, the Genome browser snapshots of other genes involved in heat tolerance should be shown.

*Reply: We added three more loci in Fig. S15 that encode heat-stress-related proteins. Details of these genes are provided in the figure legend (SI lines 103-106).*

4. Fig. S16; to better support the possibility that LVGs are enriched in SGs, thereby resulting in low gene expression variability, SG enrichment score of HVGs should be shown and compared.

*Reply: As per the reviewer’s suggestion, we added the SG enrichment score of HVGs in Fig. S16. HVGs were weaker in SG association than LVGs.*

5. Fig. 2g; what does the each bar represent? What is the difference of each bar? It is described that floral tissues exhibit stronger m6A RNA methylation and lower expression variability, and reduced expression variability in flowers diminishes the gene activation to a level below the critical point. What is the critical point? How was this critical point measured? These are poorly described.

*Reply: Fig. 2g is an illustration that summarizes the main conclusion of this study. Bar represents the expression level of each sample, which is variable in leaf and rather uniform in flower. The critical point is an arbitrary threshold required for the full impact on heat tolerance. This is now clarified in the revised figure legend (lines 496-501).*

Reviewer #3:

I thank the authors for addressing my previous concerns. However, I don't think the authors provided the Methods details in the revised manuscript. It's still not easy for me to follow how the analyses were done.

*Reply: We further detailed the descriptions of the methods in the revised manuscript which is described below, and hope it is now clear enough.*

1. The authors mentioned the custom codes is provided at https://github.com/JungnamChoLab. I actually did not find the corresponding codes used for this study.

*Reply: The codes are deposited to a folder named “RNA_Methylation”. The link to the folder is provided in the code availability statement in the Methods (https://github.com/JungnamChoLab/RNA_Methylation, line 276).*

2. Which software was used for the prediction of consensus sequence motif? How the input sequences were generated for the analysis?

*Reply: We first selected the 1,000 most significant m6A-enriched regions and RRACH sequence motif was searched in the selected regions. The identified sequences were analyzed for their frequency and visualized by weblogo tool. This is now detailed in the Methods (lines 237-240).*

**Third round of review**

**Reviewer 1**

The revised manuscript have addressed most of my comments in the last reviewing.

**Reviewer 3**

I have no further comments.
